# Boosting Zinc Hybrid Supercapacitor Performance via Thiol Functionalization of Graphene‐Based Cathodes

**DOI:** 10.1002/advs.202309041

**Published:** 2024-03-21

**Authors:** Cataldo Valentini, Verónica Montes‐García, Artur Ciesielski, Paolo Samorì

**Affiliations:** ^1^ Université de Strasbourg and CNRS ISIS 8 allée Gaspard Monge Strasbourg 67000 France; ^2^ Centre for Advanced Technologies Adam Mickiewicz University Uniwersytetu Poznańskiego 10 Poznań 61‐614 Poland

**Keywords:** energy storage devices, functional porous materials, graphene, redox active materials, zinc hybid supercapacitors

## Abstract

Zinc hybrid supercapacitors (Zn‐HSCs) hold immense potential toward the next‐generation energy storage systems, effectively spanning the divide between conventional lithium‐ion batteries (LIBs) and supercapacitors. Unfortunately, the energy density of most of Zn‐HSCs has not yet rivalled the levels observed in LIBs. The electrochemical performance of aqueous Zn‐HSCs can be enhanced through the chemical functionalization of graphene‐based cathode materials with thiol moieties as they will be highly suitable for favoring Zn^2+^ adsorption/desorption. Here, a single‐step reaction is employed to synthesize thiol‐functionalized reduced graphene oxide (rGOSH), incorporating both oxygen functional groups (OFGs) and thiol functionalities, as demonstrated by X‐ray photoelectron spectroscopy (XPS) studies. Electrochemical analysis reveals that rGOSH cathodes exhibit a specific capacitance (540 F g^−1^) and specific capacity (139 mAh g^−1^) at 0.1 A g^−1^ as well as long‐term stability, with over 92% capacitance retention after 10 000 cycles, outperforming chemically reduced graphene oxide (CrGO). Notably, rGOSH electrodes displayed an exceptional maximum energy density of 187.6 Wh kg^−1^ and power density of 48.6 kW kg^−1^. Overall, this study offers an unprecedented powerful strategy for the design and optimization of cathode materials, paving the way for efficient and sustainable energy storage solutions to meet the increasing demands of modern energy applications.

## Graphical abstract

This work presents a promising strategy for efficient and sustainable energy storage solutions in modern energy applications. Thiol‐functionalized reduced graphene oxide (rGOSH) cathodes significantly enhance the electrochemical performance of rechargeable aqueous zinc hybrid supercapacitors (Zn‐HSCs), achieving an outstanding maximum energy density of 187.6 Wh kg^−1^ and a power density of 48.6 kW kg^−1^, surpassing all reported cathode materials for Zn‐HSCs.

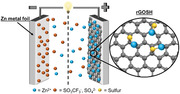

## 1 Introduction

Over the past years, lithium‐ion batteries (LIBs) and supercapacitors (SCs) have emerged as essential components for application as energy storage systems (ESS). While LIBs exhibit high energy density (100–200 Wh kg^−1^), slow charging time and low self‐discharge, SCs display a superior power density (10–20 kW kg^−1^), fast charge–discharge rate and long cycle life.^[^ ^1^, ^2^, ^3^, ^4^ ^]^ The primary limitations of LIBs concern the safety of the devices due to the highly flammable electrolytes, along with the low power density and the substantial cost of lithium (3–12k $ kg^−1^ in 2022). Furthermore, because of the limited and uneven distribution of lithium reserves on Earth, lithium has been classified as critical raw material (CRM) since 2020.^[^ ^5^, ^6^ ^]^ On the other hand, the major drawback of commercially available SCs is their low energy density when compared with LIBs. As a result, there is a growing interest within the research community in searching alternative ESS.

Aqueous hybrid supercapacitors (HSCs), which combine one capacitive‐type electrode with a battery‐type electrode in a single device, represent a promising emerging technology. By utilizing non‐flammable electrolytes, these devices could achieve remarkable energy and power densities, bridging the gap between traditional batteries and SCs.^[^ ^7^ ^]^ Among various aqueous HSCs, rechargeable aqueous zinc ion hybrid supercapacitors (Zn‐HSCs) are the most promising ones for technological applications because the use of Zn metal as anode has several advantages, such as its abundance, chemical stability, low cost (3 $ kg^−1^ in 2022), high theoretical capacity (820 mAh g^−1^), and low redox potential (−0.763 V versus standard H_2_ electrode).^[^ ^8^ ^]^ As cathodes, different materials have been explored, including carbon‐based (e.g., porous carbon, graphene, etc.), metal‐oxides, and MXenes.^[^ ^9^, ^10^ ^]^ Among them, carbon electrodes established themselves as optimal cathodes because of their low cost, high chemical stability and good conductivity. Unfortunately, the energy densities of most Zn‐HSCs using carbon‐based cathodes have not yet reached the levels observed in LIBs.^[^ ^11^ ^]^ Hence, there is a strong demand for the rational design of novel nanostructured cathode materials, exhibiting superior electrochemical performance, to render Zn‐HSCs suitable for next‐generation energy storage applications.

To increase the energy density and therefore the affinity of cathode materials for Zn^2+^ ions, different approaches have been adopted, including surface area and pore size engineering, heteroatom doping and pseudocapacitance incorporation.^[^ ^12^, ^13^, ^14^ ^]^ For instance, cathodes utilizing activated carbon (AC) or activated microwave expanded graphite oxide (aMEGO) have demonstrated remarkable performance in Zn‐HSCs. AC exhibited a large surface area of 1923 m^2^ g^−1^, leading to a high specific capacity of 121 mAh g^−1^ and excellent cycling stability (91% capacity retention after 10 000 cycles).^[^ ^15^ ^]^ On the other hand, aMEGO, with its extensive surface area of 2950 m^2^ g^−1^, achieved a specific capacitance of 166 F g^−1^ at a current density of 0.5 A g^−1^.^[^ ^16^ ^]^ The heteroatom doping strategy not only greatly enhances the chemical adsorption process of Zn^2+^ ions but it also significantly improves the conductivity, surface wettability, and active sites of the pristine materials. For example, Lu et al. developed an aqueous Zn‐HSC utilizing hierarchically porous carbon treated with ammonia as cathode material. The nitrogen doping enabled the material to achieve a specific capacity of 180 mAh g^−1^ at current density of 5 A/g in contrast to the undoped material which exhibited a specific capacity of only 70 mAh g^−1^.^[^ ^17^ ^]^ Besides, Qui et al. demonstrated that a cathode material based on nitrogen, and boron co‐doped porous carbon can reach specific capacities of 127.7 mAh g^−1^ at a current density of 0.5 A g^−1^ and exceptional energy and power densities of 86.8 Wh kg^−1^ and 12.2 kW kg^−1^, respectively, in Zn‐HSC device.^[^ ^18^ ^]^ Finally, it has been shown that the incorporation of various active sites (e.g., oxygen functional groups (OFGs), N‐atom rich groups, or sulfur‐based functional groups) imparts pseudocapacitance to the pristine materials and has a great impact in the overall electrochemical performance.^[^ ^19^, ^20^ ^]^ Li et al studied a nitrogen and phosphorus co‐doped graphene‐based material as cathode in Zn‐HSC reaching a specific capacitance of 210 F g^−1^ at a current density of 0.5 A g^−1^ with a maximum energy density of 94.6 Wh kg^−1^ and a large power density of 4500 W kg^−1^.^[^ ^21^ ^]^ A nitrogen and sulfur co‐doping of rGO blended with polyaniline was achieved by Hou et al by a stepwise approach starting from an hydrothermal threating of GO with thiourea followed by an in situ aniline polymerization (i.e., N,S‐rGO/PA). The N,S‐rGO/PA obtained showed a specific capacitance of 268 F g^−1^ at 0.1 A g^−1^ and a cyclability of 93% capacitance retention over 10 000 cycles at a current density of 5 A g^−1^^[^ ^22^ ^]^. Shao et al. showed that enhancing the Zn‐HSC performance using reduced graphene oxide (rGO)‐based cathodes can be achieved by selectively introducing OFGs onto the rGO (HHT‐rGO) surface through controlled hydrothermal oxidation with hydrogen peroxide. The incorporation of OFGs provided additional Zn^2+^ active sites and improved the wettability of the pristine material, thereby facilitating the infiltration of the electrolyte within the active material. The resulting HHT‐rGO electrode showed a specific capacitance of 277 F g^−1^, and a high cycling stability of 97.8% capacitance retention after 20 000 cycles in 1 m ZnSO_4_ electrolyte.^[^ ^23^ ^]^ Recently, we have demonstrated that the incorporation of thiol groups in the chemical structure of covalent organic frameworks (COFs) can boost their performance as electrochemical SCs, achieving an areal capacitance of 118 mF cm^−2^ and a capacitance retention >95% after 1000 cycles.^[^ ^24^ ^]^ While the incorporation of OFGs has been widely explored as a strategy to improve the electrochemical performance of rGO‐based materials, the incorporation of sulfur‐based functional groups remains unexplored. According to previously reported studies on oxophilicity and thiophilicity of metal ions, Zn^2+^ ions possess a higher affinity for sulfur‐based groups than OFGs.^[^ ^25^ ^]^ The reversible supramolecular interaction between Zn^2+^ ions and thiol groups at the cathode surface would favor the adsorption/desorption of the electrolyte ions during the charge–discharge cycles, enhancing the performance of the Zn‐HSCs.

Herein, we report for the first time, the synthesis of thiol functionalized reduced graphene oxide (rGOSH) via one pot reaction, where GO reduction and thiol functionalization occur simultaneously under mild conditions. An aqueous Zn‐HSC is assembled, in which rGOSH and Zn foil are used as cathode an anode, respectively, while an aqueous solution containing zinc trifluoromethanesulfonate (Zn(CF_3_SO_3_)_2_) serves as electrolyte. X‐ray photoelectron spectroscopy (XPS) showed that both, OFGs and thiol functionalities are present on the rGOSH surface. While both functionalities act as active coordination sites, the higher affinity of the thiol groups for Zn^2+^ ions reduces the chemical adsorption barrier of Zn ions on rGO‐based cathode, favoring the redox processes at electrode interface.^[^ ^25^ ^]^ The superior electrochemical performance of rGOSH as cathode material in aqueous Zn‐HSCs is correlated with various structural parameters, including surface area, pore size, electronic conductivity and amount, and nature of active sites in comparison with CrGO, thereby providing guidelines for the facile fabrication of high‐performance Zn hybrid supercapacitors.

## 2 Results and Discussion

### 2.1 Structural Characterization

**Figure 1:**
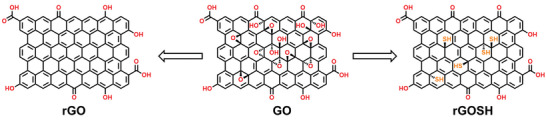
Synthetic pathway of CrGO and rGOSH.

The synthesis of rGOSH is performed by using a modified reported procedure by Rourke et al., see Experimental Section for details, where potassium thioacetate acts both as reducing agent as well as a sulfur source (**Figure** **1**).^[^ ^26^ ^]^ Specifically, a degassed mixture of GO and potassium thioacetate in dimethyl sulfoxide (DMSO) is heated to 50 °C for 16 h. Afterward rGOSH is collected by centrifugation and washed thoroughly prior to lyophilization. On the other hand, CrGO is obtained by following our recently reported procedure by using hydrazine monohydrate (N_2_H_4_) as reducing agent and 12 h of reaction time (Figure 1).^[^ ^27^ ^]^

**Figure 2:**
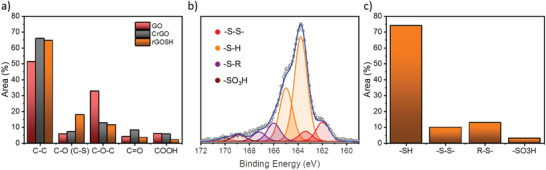
XPS analysis: a) Relative contribution of C1s peak components estimated by dividing the area under each component by the whole C1s peak, b) high‐resolution S2p peaks of rGOSH, and c) relative contribution of S2p peak components estimated by dividing the area under each component by the whole S2p peak.

To gain insights into the chemical composition of rGOSH and CrGO, X‐ray photoelectron spectroscopy (XPS) analysis is first performed (**Figure** **2**; Figures S1 and S2, Supporting Information). From the XPS survey spectra (Figure S1, Supporting Information), the C/O ratio is estimated to determine the degree of GO reduction. While in GO the C/O ratio amounts to 2.54, it increases substantially after its reduction and/or functionalization, obtaining values of 12.79 and 5.19 for CrGO and rGOSH, respectively. The significant increase of C/O ratio is a first proof that the majority of the OFG in the carbon plane are removed and the π‐conjugation is largely restored.

The high‐resolution C1s spectra are fitted using 5 Gaussian–Lorentzian curves for the five chemical environments of GO, CrGO and rGOSH: 284.4 eV C─C, 285.15 eV C─O/C─S, 286.48 eV C─O─C, 287.38 eV C═O, and 288.50 eV COOH (Figure S2a,c, Supporting Information).^[^ ^28^ ^]^ Figure 2a shows the relative contribution of C1s peak components for GO, CrGO and rGOSH. It reveals a significant increase in the content of C─C bond (51% for GO) for CrGO (66%) and rGOSH (65%). Conversely, the peak area corresponding to C─O─C, C═O, and COOH bonds decreases for both CrGO and rGOH. In particular, the C─O─C peak area drops significantly from 35% (GO) to 11% in both CrGO and rGOSH. In full agreement with our previous report, the chemical reduction is accompanied by an increase in the peak area associated with C─O, as the content of C_sp_^2^─O─C_sp_^2^ grows in the first steps of the reduction.^[^ ^28^ ^]^ For rGOSH, the peak area associated with C─O/C─S shows a notably higher increase (up to 18%) due to the additional contribution of thiol functionalization.

Likewise, the high resolution O1s spectra (Figure S2b,d, Supporting Information) are fitted with three Gaussian–Lorentzian curves at 531.0 eV C═O, 532.0 eV C_sp_^3^─O and 533.3 eV C_sp_^2^─O.^[^ ^28^ ^]^ For rGOSH, the peak area associated of these curves amounts to 22%, 30%, and 48%, respectively. Interestingly this demonstrates the coexistence of oxygen functional groups together with thiol moieties in rGOSH, where both can act as active sites for the coordination with Zn^2+^ ions.

The XPS survey of rGOSH reveals an atomic percentage of sulfur of ≈4%, showcasing the successful sulfur functionalization of the graphene basal plane (Figure S1, Supporting Information). To ascertain the chemical state of sulfur on rGOSH, the high‐resolution XPS analysis of the S2p peak (Figures 2b,c) is then performed. Figure 2b reveals that the dominant peak corresponds to ─SH thiol groups, which is approximately 74% of the sulfur species. Two smaller peaks are also observed, corresponding to ─S─S─ disulfide moiety (10%), and R‐S‐ alkyl sulfide group (13%). Furthermore, a minor peak corresponding to ─SO_3_H groups (3%) is also present, which could be attributed to traces of ─SO_3_H already present in the GO precursor.

Thermogravimetric analysis (TGA) is then performed to assess the thermal stability of CrGO and rGOSH. Figure S3 (Supporting Information) shows that the thermal stability of GO is increased upon chemical reduction and the Td_10_ (thermal decomposition of 10% weight) increases from 70 °C (pristine GO) to 114 °C (CrGO) and to 206 °C (rGOSH). The flaky structure remains unaltered during the reduction process, as evidenced from the scanning electron microscopy (SEM) images (Figure S4, Supporting Information) of both CrGO and rGOSH. The elemental analysis from energy‐dispersive X‐ray (EDX) spectroscopy (Figures S5 and S6, Supporting Information) reveals the presence solely of C and O elements in CrGO and C, O, and S elements in rGOSH. Importantly, these elements are homogeneously distributed on both CrGO and rGOSH as revealed in the EDX mappings of Figure S6 (Supporting Information).

Raman spectroscopy provides valuable insights into the chemical composition and structural parameters of rGO‐based materials.^[^ ^29^, ^30^ ^]^ The Raman spectra of GO, rGOSH and CrGO are deconvoluted using five Lorentzian curves representing first‐order Raman modes (D, Dʹʹ, Dʹ, D*, and G) (Figure S7, Supporting Information). The D band (≈1350 cm^−1^) corresponds to breathing modes of photons of A_1g_ symmetry, while the G band (≈1585 cm^−1^) relates to first‐order scattering of E_2g_ phonons of the sp^2^ carbon structure.^[^ ^30^ ^]^ The I_D_/I_G_ ratio, which is an insightful parameter to estimate the degree of reduction in GO derivatives, increases for both CrGO and rGOSH, indicating restoration of sp^2^ conjugation due to the removal of oxygen functional groups from GO.^[^ ^28^ ^]^ Additional bands (D′′, D*, and D′) arise from the defects present in the graphitic structure of the carbon material.^[^ ^29^, ^30^, ^31^ ^]^ Although ideally, these ratios should decrease with the reduction degree, the slight increase in I_D_’/I_G_, I_D’’_/I_G_, and I_D*_/I_G_ ratios in most cases suggests that the reduction process is creating defects in the graphitic structure of the carbon material.

The effect of reduction and thiol functionalization on the crystallinity of rGOSH is investigated by powder X‐Ray diffraction (PXRD) (Figure S8 and Table S2, Supporting Information). The diffraction pattern of rGOSH displays a prominent peak at 2θ ≈24° (002 plane) with a broad FWHM of ≈7.5°. Additionally, the peak at 2θ ≈24° exhibits a FWHM nearly five times larger than the small peak at 2θ ≈14°, which indicates a smaller crystallite size compared to pristine GO. Crystallite thickness, d‐spacing, and the number of layers are determined (Table S2, Supporting Information), and the values obtained for rGOSH closely resemble those of our previously reported CrGO.^[^ ^28^ ^]^ This confirms that our reduction and functionalization protocol does not significantly alter the crystallinity of the resulting rGOSH.

**Figure 3:**
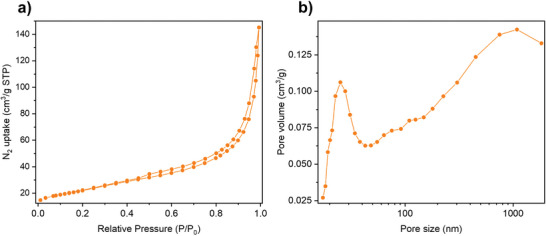
a) Nitrogen adsorption–desorption isotherms and b) pore size distribution of rGOSH

The specific surface area and average pore size of rGOSH is evaluated by recording N_2_ adsorption–desorption isotherms at 77 K (Figure **3**). The calculated Brunauer–Emmett–Teller (BET) surface area of rGOSH amounts to 78.96 m^2^ g^−1^, indicating a sixfold increase in the surface area when compared to pristine GO (12.61 m^2^ g^−1^) yet being slightly smaller than CrGO (124.92 m^2^ g^−1^) (Table S3, Supporting Information).^[^ ^32^ ^]^ Interestingly, the average pore size of rGOSH amounts to 10.2 nm, thus it is nearly twice the one of GO or CrGO. Figure S10 (Supporting Information) shows the differential distribution of pore volumes versus pore sizes for rGOSH, revealing that rGOSH has a predominant mesopore distribution with sizes in the range between 10 and 100 nm. The electrochemical performance of electrode materials is influenced by various key physical characteristics, including surface area, porosity, conductivity, ion accessibility, and chemical stability, among others. In our study, we have reported these parameters for GO, CrGO, and rGOSH in Table S3 (Supporting Information). Despite GO's abundance of active groups, its low specific surface area translates to comparatively inferior electrochemical performance when compared to CrGO and rGOSH. Notably, while CrGO and rGOSH exhibit similar surface areas, the enhanced average pore size and presence of thiol groups in rGOSH are pivotal factors contributing to its superior performance over CrGO.

To cast further light onto the physical properties of CrGO and rGOSH, their electrical performance has been assessed by thin film conductivity measurements. Pellets of the different materials have been prepared (see Experimental Section) and the film resistivity was measured with a four‐point probe (FPP). The film resistivity of pristine GO material, due to its insulating character, resulted above the detection limit of our instrument. Conversely, the measured film conductivity of CrGO and rGOSH amounted to 3848 ± 21 and 60 ± 2 S m^−1^, respectively (Table S3, Supporting Information). Although the electrical conductivity of rGOSH is substantially lower than the one of CrGO, we have previously observed that an electrical conductivity of at least 20 S m^−1^ is required to achieve high electrochemical performance in energy storage devices incorporating rGO‐based electrode materials, yet higher electrical conductivity values do not lead to a further performance enhancement.^[^ ^28^ ^]^

### 2.2 Electrochemical Analysis

**Figure 4:**
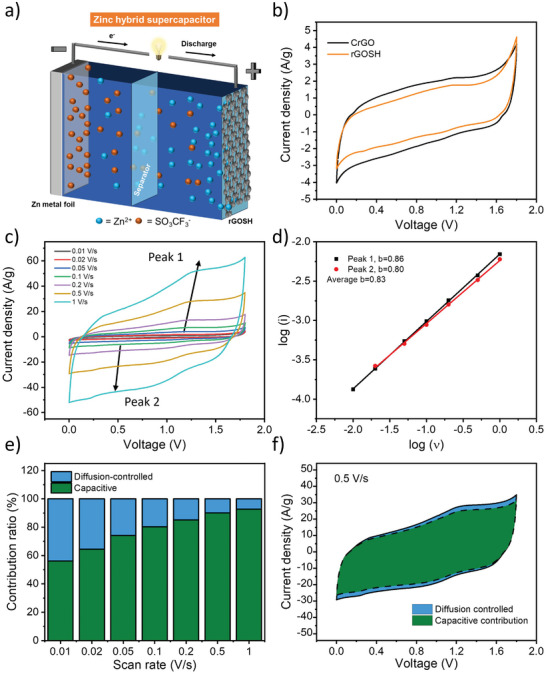
a) Scheme of rGOSH || Zn(CF_3_SO_3_)_2_ || Zn energy storage system, b) CV curves of CrGO (black curve) and rGOSH (orange curve) at 0.2 V s^−1^, c) CV curves of rGOSH at various scan rates, d) fitting plots between log(i) and log (υ) at various peak currents, e) capacitive (contribution) and diffusion‐controlled contribution of rGOSH at various scan rates, and f) capacitive (contribution) and diffusion‐controlled contribution fraction for the CV curve at 0.5 V s^−1^ of rGOSH.

The electrochemical performance of CrGO and rGOSH in Zn^2+^ storage is assessed in a two‐electrode configuration by assembling a CrGO or rGOSH cathode with a Zn foil anode and 4 m zinc trifluoromethanesulfonate (Zn (CF_3_SO_3_)_2_) aqueous electrolyte (**Figure** **4**a). Figure 4b displays the cyclic voltammograms (CV) of CrGO and rGOSH between 0 and 1.8 V at a scan rate of 0.2 V s^−1^. Both CrGO and rGOSH exhibit quasi‐rectangular CV curves with no prominent redox peaks, evidence of their pseudocapacitive or hybrid behavior. Figure 4c and Figure S9a (Supporting Information) show the CV profiles of rGOSH and CrGO, respectively, at different scan rates. The symmetrical capacitive behavior is sustained even at an ultrafast scan rate of 1 V s^−1^, indicating rapid charge propagation within the electrode material and excellent rate capability. In order to gain insight into the charge storage mechanism of rGO‐based materials, the kinetics of the electrochemical processes occurring at both electrode materials are analyzed using the procedure proposed by Wu et al. (see Figure 4d; see Figure S9b, Supporting Information, for rGOSH and CrGO, respectively).^[^ ^33^ ^]^ The relation of the peak current (i) and scan rate (ν) from CV complies with the equation:

(1) $$i=a\nu^{b}$$

In the given equation, *a* and *b* are adjustable parameters. Typically, the coefficient *b* falls within the range of 0.5–1.0. A *b*‐value of 0.5 indicates a diffusion‐limited electrochemical process, while a *b*‐value of 1.0 suggests a capacitive‐limited process. The average values of b for CrGO and rGOSH are 0.84 and 0.83, respectively, indicating that both rGO‐based materials possess a hybrid nature with a charge storage mechanism predominantly ruled by a capacitive contribution and partial diffusion‐controlled contribution. To further analyze the capacitive contribution and the diffusion‐controlled contribution at a specific scan rate, the Equation (1) can be divided into two parts, as shown below:

(2) $$i=k_{1}\nu +k_{2}\nu^{1/2}$$

where *k*_1_ *ν* and *k*_2_ *ν* ^1/2^ represent the capacitive and diffusion limited effects, respectively. The capacitive and diffusion‐controlled capacity values at different rates are calculated and shown in Figure 4e and Figure S12 (Supporting Information) for rGOSH and Figures S10 and S11 (Supporting Information) for CrGO. At a scan rate of 0.01 V s^−1^, around 56.2% of the total current in rGOSH is governed by capacitive‐limited processes. As the scan rate increases, the contribution ratio of the capacitive process gradually rises for both rGOSH and CrGO materials (Figure 4e; Figure S10, Supporting Information). Notably, at the highest scan rate (1 V s^−1^), rGOSH exhibits the highest capacitive contribution of 92.77% to the charge storage mechanism. Consequently, the coexistence of capacitive and diffusion‐controlled charge storage mechanisms confirms the hybrid nature of CrGO and rGOSH materials, providing notable advantages for energy storage applications. Electrochemical impedance spectroscopy (EIS) data is analyzed using Nyquist plots (Figure S13, Supporting Information) and the experimental results are well fitted with the indicated circuit (Figure S14, Supporting Information). Both rGO‐based materials exhibit a low charge transfer resistance (Rct) (23.59 Ω for CrGO and 46.41 Ω for rGOSH) providing evidence for their high electrical conductivity and rate capability (Table S4, Supporting Information).

**Figure 5:**
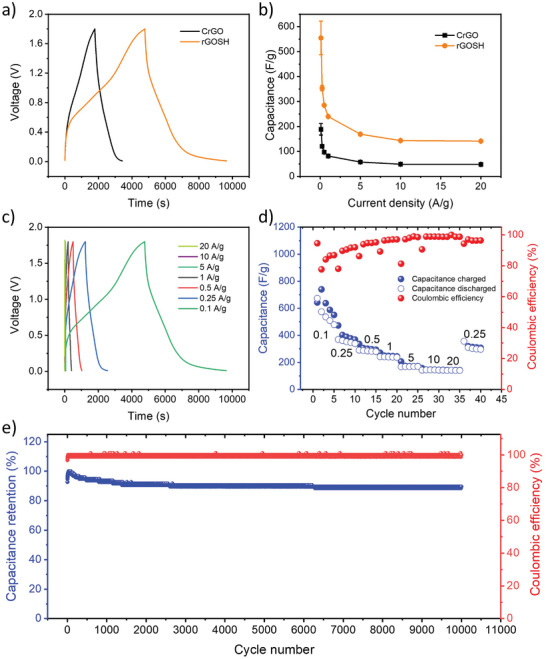
a) GCD profiles of CrGO (black curve) and rGOSH (orange curve) at 0.1 A g^−1^, b) specific capacitance of CrGO (black curve) and rGOSH (orange curve) at various current densities, c) GCD profiles of rGOSH at various current densities, and d) specific capacitance charged (blue points), discharged (white points) and coulombic efficiency (red points) of rGOSH at various current densities. Each current density is express in A g^−1^, e) long‐term cycling performance of rGOSH at 1 A g^−1^.

The galvanostatic charge/discharge (GCD) profiles of CrGO and rGOSH electrodes at a current density of 0.1 A g^−1^ are shown in Figure **5**a. The voltage–time curve exhibits a quasi‐linear shape, with no evident plateaus, confirming the hybrid nature of CrGO and rGOSH materials. The specific capacitances of CrGO and rGOSH electrodes are calculated from GCD curves at different current densities, as monitored in Figure 5b (see Experimental Section for calculation details). The maximum specific capacitance amounts to 540 ± 48 and 260 ± 10 F g^−1^ for rGOSH and CrGO, respectively. In our previous systematic study on thermally reduced GO (TrGO) and CrGO, we have shown that crucial properties such as surface area, porosity, electrical and ionic conductivity, and electrochemical activity significantly affect the electrochemical performance of materials used in ESS.^[^ ^32^ ^]^ In the present case, the electrochemical performance of rGOSH electrodes is more than twice the performance of CrGO electrodes, despite the superior electrical conductivity of the latter. While their surface area is comparable, the main differences between the two materials are the higher porosity of rGOSH, together with the nature and amount of active sites, proving that the combination of OFGs and thiol groups is an excellent solution for boosting the electrochemical performance of rGO materials for Zn^2+^ ions storage.

The GCD profiles of rGOSH (Figure 5c; Figure S16a, Supporting Information) in the explored potential window reveal the absence of Ohmic drop (IR drop) and a high capacitance (Figure 5d) of 540 ± 48, 353 ± 15, 284 ± 2, 240 ± 1, 169 ± 0.5, 144 ± 0.6, and 141 ± 0.3 F g^−1^ at a current density of 0.1, 0.25, 0.5, 1, 5, 10, and 20 A g^−1^, respectively, which are superior to CrGO (Figures S15a and S17b, Supporting Information) and to most of the reported cathodes based other organic and inorganic electrode materials in Zn‐HSCs (Table S8, Supporting Information). Significantly, these capacitance values represent the average of five devices, emphasizing the statistical robustness of our electrode fabrication methodology. In particular, looking at other reported heretoatom functionalized rGO, the N,S‐rGO/PA, N,P‐rGO, and HHT‐rGO show specific capacitances that are 52%, 26%, and 50%, respectively, lower than the one we obtained for rGOSH (Table S8, Supporting Information). Even after undergoing charge/discharge cycles at an extremely high current density of 20 A g^−1^, a substantial capacitance of 313 F g^−1^ is achieved at 0.25 A g^−1^. Additionally, as both CrGO and rGOSH are classified as hybrid materials, specific capacity is also calculated from the GCD profiles, obtaining a state‐of‐the‐art capacity of 139 mAh g^−1^ at 0.1 A g^−1^ for rGOSH.

Long‐term stability of the prepared rGO‐based electrodes is then investigated using galvanostatic charge–discharge cycles at the current density of 1 A g^−1^ (see Figure 5e; see Figure S19, Supporting Information, for rGOSH and CrGO, respectively). While CrGO electrodes exhibit a drop of capacitance to 70% after 500 charge/discharge cycles and kept constant for 10 000 cycles, rGOSH electrodes show an excellent long‐term stability with a capacitance retention higher than 92% over 10 000 cycles, together with a Coulombic efficiency of nearly 100%, indicating the better reversible supramolecular interaction between thiol groups and Zn^2+^ ions (Figure 5e; Figure S19, Supporting Information).

Remarkably, the Ragone plot of the prepared rGO‐based electrodes (Figure S20, Supporting Information) displays an outstanding maximum energy density of 187.6 Wh kg^−1^ together with an excellent power density of 48.61 kW kg^−1^ for rGOSH electrodes, which is within the state of the art of previously reported cathode materials for Zn‐HSCs (Figure S20 and Table S8, Supporting Information). In particular, rGOSH demonstrates a power density 140% higher than the one of activated carbon (AC). Moreover, as can be seen in Table S8 (Supporting Information), the maximum areal energy and power densities for rGOSH amount to 158.62 µWh cm^−2^ and 4.5 mW cm^−2^, respectively, significantly surpassing those of AC (115.4 µWh cm^−2^ and 3.9 mW cm^−2^, respectively), proving that thiol functionalization represents a viable strategy to obtain devices with high energy and power density.

Additionally, in order to demonstrate that active sites thiol groups are involved in the charge storage mechanism of Zn^2+^ ions, ex situ XPS analyses were performed in the pristine, discharged (at 0 V) and charged (at 1.8 V) states of rGOSH (Figure S21a, Supporting Information). The high‐resolution Zn2p spectra (Figure S21b, Supporting Information) reveal the typical peaks at 1021 and 1044 eV of Zn^2+^, which exhibit a higher intensity in the discharged state as a consequence of their coordination with the rGOSH electrodes. Moreover, extensive electrochemical analysis (e.g., 10 000 charge/discharge cycles) confirms the highly reversible nature of Zn^2+^ storage within rGOSH. In order to assess which active groups (i.e., carbonyl, hydroxyl and thiol) have a higher affinity for Zn^2+^ ions, the corresponding binding energies are calculated using Gaussian software (Figure S22 and Tables S5–S7, Supporting Information). The following equation is employed:

(3) $$E_{\mathrm{bind}}=E_{\mathrm{opt}-\mathrm{dimer}}\;-\left(E_{\mathrm{opt}-\mathrm{mon}1}+E_{\mathrm{opt}-\mathrm{mon}2}\right)$$

Where *E_bind_* is the resulting binding energy, *E*_*opt* − *dimer*_, the optimized energy of the complex and *E*_*opt* − *mon*1_ and *E*_*opt* − *mon*2_ are the optimized energy of the monomer 1 and 2, respectively.

As can be seen in Table S7 (Supporting Information), the higher binding energy between SH groups and Zn ions (i.e., −190.44 kcal mol^−1^) implies a more stable interaction, facilitating better retention of Zn ions during the charge–discharge process, potentially leading to higher capacitance/capacity, enhanced cycling stability, and improved overall efficiency in energy storage applications

## 3 Conclusion

In summary, this study highlights the significance of developing novel nanostructured cathode materials for improving the energy density and electrochemical performance of rechargeable aqueous Zn‐HSCs. The synthesis of rGOSH through a one‐pot reaction, where reduction and thiol functionalization occur simultaneously under mild conditions, allows for the incorporation of both OFGs and thiol functionalities on the surface of the material, as shown by XPS analysis. The comparison between CrGO and rGOSH demonstrates the latter's superiority as a promising cathode material for next‐generation energy storage applications. Specifically, rGOSH shows a high specific capacitance (540 F g^−1^) and specific capacity (139 mAh g^−1^), both at 0.1 A g^−1^, as well as outstanding capacitance retention (>92%) over 10 000 cycles, indicating excellent long‐term stability compared to CrGO. The maximum energy density of 187.6 Wh kg^−1^ achieved by rGOSH is remarkable and greater than previously reported cathode materials for Zn‐HSCs. Additionally, rGOSH showed a high‐power density of 48.6 kW kg^−1^, further confirming its potential for high‐performance energy storage applications. The combination of OFGs and thiol groups provides a unique hybrid nature that significantly contributes to the overall superior performance of rGOSH. All in all, this study sheds light onto the promising advancements in energy storage technology, offering an innovative approach to address the challenges associated with current energy storage systems. The development of rGOSH opens up new avenues for the design and optimization of materials to meet the increasing demands for high‐energy and high‐power storage devices, paving the way toward more sustainable and efficient energy storage solutions. The development of rGOSH introduces an exciting era in material design and enhancement, responding to the escalating need for energy storage systems with superior power and energy capacities. Its thiol functionalization broadens its scope beyond energy storage to catalysis, sensing devices, biomedical applications, surface modification, and wastewater treatment, exhibiting remarkable versatility across diverse applications. This transformative material promises an extensive range of functionalities, heralding a new era in multifaceted applications.

## 4 Experimental Section

### Materials

Graphene oxide (GO, 4 mg mL^−1^, monolayer content >95%, Graphenea), dimethyl sulfoxide (DMSO), potassium thioacetate, hydrochloric acid, ethanol, acetone, diethyl ether hydrazine monohydrate (N_2_H_4_), *N*‐methyl‐2‐pyrrolidone (NMP), zinc foil (thickness of 0.25 mm) (Sigma Aldrich), and zinc trifluoromethanesulfonate (Sigma Aldrich), polyvinylidene difluoride (PVDF) (MTI), conductive carbon black Super P (H30253) (Alfa Aesar), and coin cells cases (S4R).

### GO (Powder)

Solid GO was produced by lyophilization from the commercially available GO solution using a freeze‐dryer (Christ).

### Synthesis of rGOSH

rGOSH was prepared by modifying a reported protocol.^[^ ^26^ ^]^ GO (100 mg) was dispersed in DMSO (50 ml) via sonication and degassed bubbling N_2_ for 1.5 h. Potassium thioacetate (70 mg) was added and the solution degassed with N_2_ for 30 min. The mixture was then heated to 50 °C for 16 h. After cooling down,HCl (1 m, 5 ml) was added to the rGOSH mixture, which was then immediately centrifuged, and the solids collected. The solid was washed with acetone (20 min, x3), diethyl ether (10 min, x2) and distilled water (2 h, x3) before being lyophilized to yield a flaky black powder, rGOSH (49 mg).

### Synthesis of CrGO

An aqueous dispersion of 30 mL of GO 10 mg mL^−1^ was diluted in 270 mL of Milli Q water and sonicated for 20 min in an ultrasonic bath cleaner (140 w). Subsequently, hydrazine monohydrate (N_2_H_4_) (final concentration of 8 g L^−1^) was added, and the pH adjusted to 9–10 with 5 wt% K_2_CO_3_. The reaction mixture was stirred for 12 h at 95 °C. The chemically reduced GO (CrGO) was collected by filtration and washed thoroughly with deionized water and ethanol. The black precipitate was then freeze‐dried for 72 h under vacuum.

### Methods

The composition, structure, and texture properties of materials were investigated by powder X‐ray powder diffraction (PXRD) patterns (Bruker D8 X‐ray diffractometer). Thermogravimetric Analyzer (TGA) decomposition curves were recorded in the range 25–300 °C operating under air or nitrogen atmosphere, with a thermal step of 10 °C min^−1^ on a Mettler Toledo TGA/SDTA851e system. X‐ray Photoelectron Spectroscopy (XPS) (Thermo Scientific K‐Alpha X‐ray photoelectron spectrometer) equipped with an aluminum X‐ray source (energy 1.4866 keV) at a vacuum level of 10^−8^–10^−9^ mbar in the main chamber. The spot size of the X‐ray beam was fixed at 400 µm. Raman spectra were acquired with a Renishaw InVia Reflex system. The spectrograph used a high‐resolution grating (2400 grooves cm^−1^) with additional bandpass filter optics, a confocal microscope, and a 2D‐CCD camera. The excitation was carried out using a 532 nm laser excitation beam, with a 100× objective, 0.2 mW maximum power and 1 s acquisition time. The specific surface area was measured using a Micromeritics ASAP 2050 surface area and porosity analyzer. Before the Brunauer–Emmett–Teller (BET) measurements, the samples were outgassed for 12 h at 95 °C. Adsorption isotherms were calculated for nitrogen adsorption at 77 K and pressure up to 1 bar. Scanning Electron Microscopy (SEM) images and Energy‐dispersive X‐ray spectroscopy (EDX) were recorded with a FEI Quanta FEG 250 instrument S3 (FEI corporate, Hillsboro, Oregon, USA).

### Four‐Point Probe Measurements

Electrical conductivity measurements were conducted on pelletized samples: 50 mg of the different materials were pressed under 10 tons with a Specac press machine. Films resistivity were measured with Jandel, Model RM3000, limit of detection 10^7^ Wsq^−1^. The resistivity (r) was obtained:

(4) $$\rho \;=\;R_{s}\cdot l$$

where *R*_s_ is the sheet resistance and *l* is thickness of the film.

### Fabrication of Zinc Hybrid Supercapacitors (Zn‐HSCs)

Zinc foil was directly used as an anode electrode after being polished with gauze and pouched into electrodes with a 8 mm diameter. The cathode electrode is composed of 90 wt% of rGOSH/CrGO, and 10 wt% of polyvinylidene difluoride (PVDF)) as the binder. N‐Methyl‐2‐pyrrolidone (NMP) solvent was added to the above mixture and then the mixture was coated onto carbon paper electrodes. The electrodes were dried in a vacuum oven at 80°C overnight. Zn‐HSCs were assembled with the electrolyte of 4 m Zn(CF_3_SO_3_)_2_ aqueous solution, a Nonwoven separator MPF30AC as a separator, and a coin battery shell. For each electro the mass loading was 1 mg cm^−2^ and the weight ratio of 90 wt.% of active material.

### Electrochemical Characterizations and Calculations of Aqueous Zn‐HSCs

The electrochemical performance of Zn‐HSCs was studied using cyclic voltammetry (CV), and electrochemical impedance spectroscopy (EIS) on Autolab PGSTAT128N Potentiostat/Galvanostat instruments with a Metrohm Autolab DuoCoin Cell Holder (Metrohm AG) at room temperature. CV was performed at scan rates of 0.01–1 V s^−1^ in the voltage range between 0 and 1.8 V. EIS measurement was recorded with a frequency range of 0.01 Hz to 1 MHz. The galvanostatic charge–discharge (GCD) tests were carried out on Neware Battery Tester (BTS‐4008T‐5 V/10 mA, Neware Technology Company, Guangdong, China). GCD curves were tested at current densities ranging from 0.1 to 1 A g^−1^.

From charge–discharge measurements, the specific capacitances (Cp), energy densities (E) and power densities (P) of CrGO and rGOSH were obtained from the acquired data using following equations^[^ ^34^, ^35^, ^36^ ^]^:

(5) $$Cp\;=\frac{2\cdot I\cdot \Delta \;t}{m\cdot \Delta V}$$

(6) $$E\;=\frac{1}{2\cdot m}\;\cdot \int_{0}^{t}V\cdot I\cdot \Delta t$$

(7) $$P\;=\frac{E}{\Delta t}$$

where I is the discharge current (A), Δt is the discharge time (s), m is the weight of the active material in an individual electrode (g), and ΔV is the discharge voltage (V) excluding the internal resistance (iR) drop during the discharge process.

## Conflict of Interest

The authors declare no conflict of interest.

## Supporting information

Supporting Information

## Data Availability

The data that support the findings of this study are available from the corresponding author upon reasonable request.
