# Supplementary material for: Boosting Zinc Hybrid Supercapacitor Performance via Thiol Functionalization of Graphene‐Based Cathodes
Source: Adv Sci (Weinh). 2024 Mar 21;11(22):2309041. doi: 10.1002/advs.202309041 (PMC11165479; doi:10.1002/advs.202309041)
Supplement: Supplementary file 1 — Supporting Information [file ADVS-11-2309041-s001.pdf]

ADVANCED SCIENCE
Open Access

Supporting Information

for *Adv. Sci.*, DOI 10.1002/advs.202309041

Boosting Zinc Hybrid Supercapacitor Performance via Thiol Functionalization of Graphene-Based Cathodes

Cataldo Valentini, Verónica Montes-García, Artur Ciesielski and Paolo Samorì**

Supporting Information

Boosting Zinc Hybrid Supercapacitor Performance via Thiol Functionalization of Graphene-Based Cathodes

Cataldo Valentini, Verónica Montes-García, Artur Ciesielski, and Paolo Samorì**

Table of contents

1. **XPS characterization** 2
2. **TGA characterization** 3
3. **SEM characterization** 3
4. **EDX characterization** 4
5. **Raman characterization** 5
6. **PXRD characterization** 5
7. **BET characterization** 7
8. **Electrochemical characterization** 8
9. **Charge – Discharge Mechanism investigation** 17
10. **Computational methods** 17
11. **References** 22

1. XPS characterization

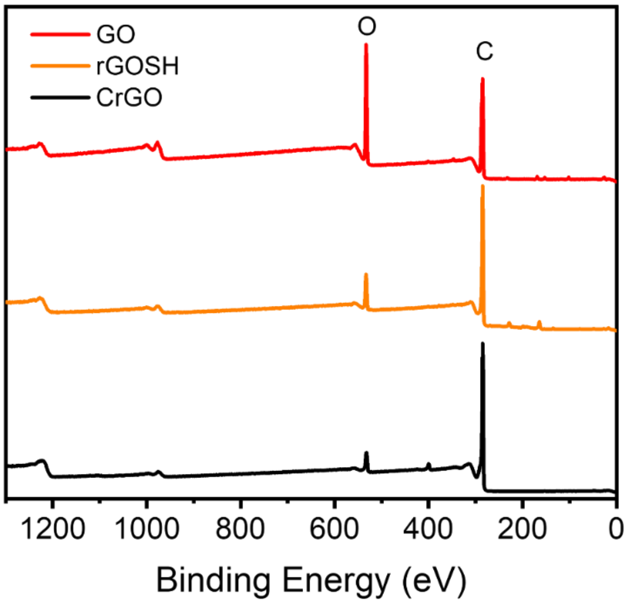

Figure S1. XPS survey spectra of GO (red curve) CrGO (black curve) and rGOSH (orange curve).

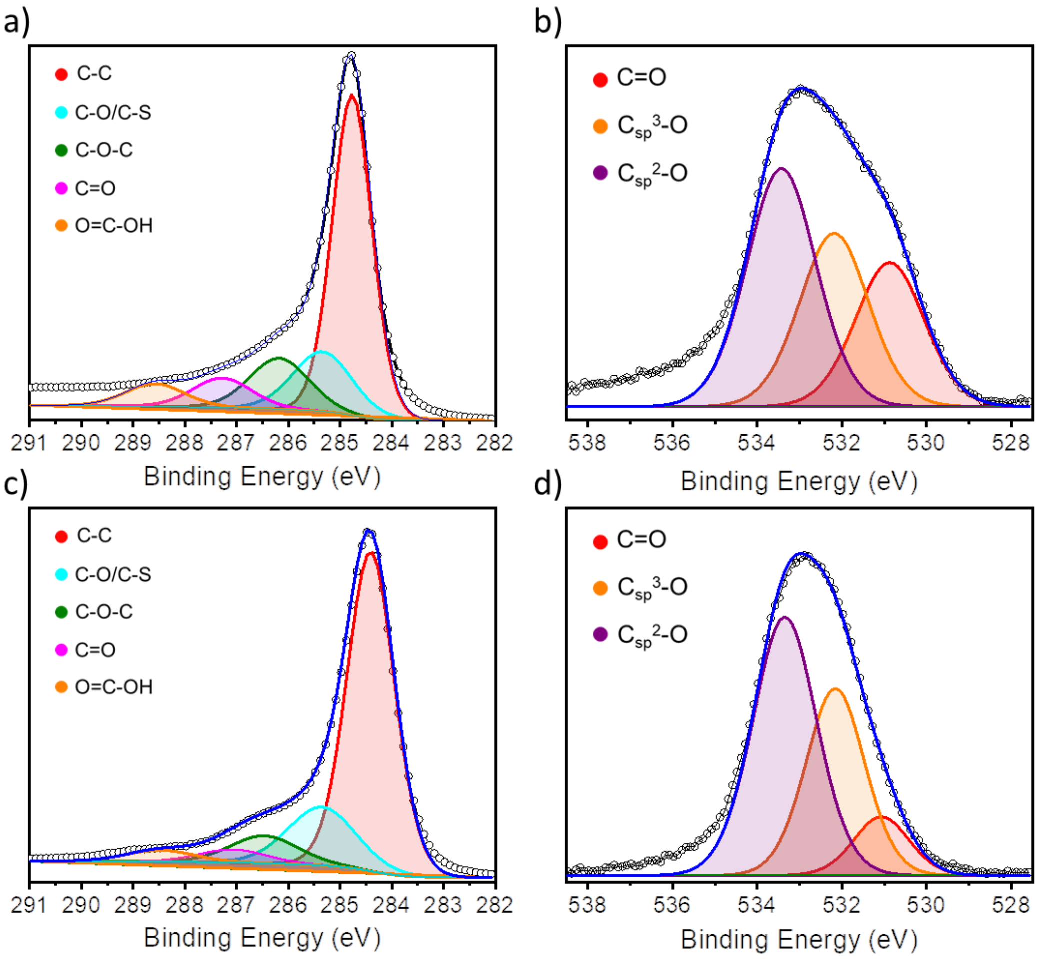

Figure S2. Fitted XPS a, c) C1s, and b, d) O1s spectra of a-b) CrGO, and c-d) rGOSH.

2. TGA characterization

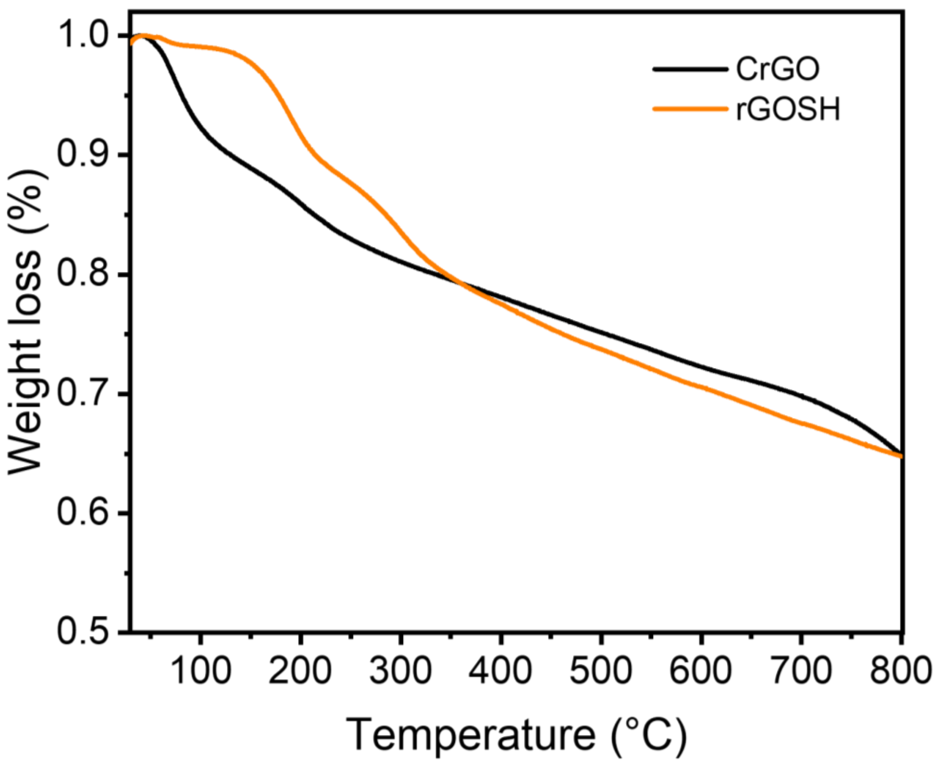

Figure S3. TGA diagram of CrGO (black curve) and rGOSH (orange curve).

3. SEM characterization

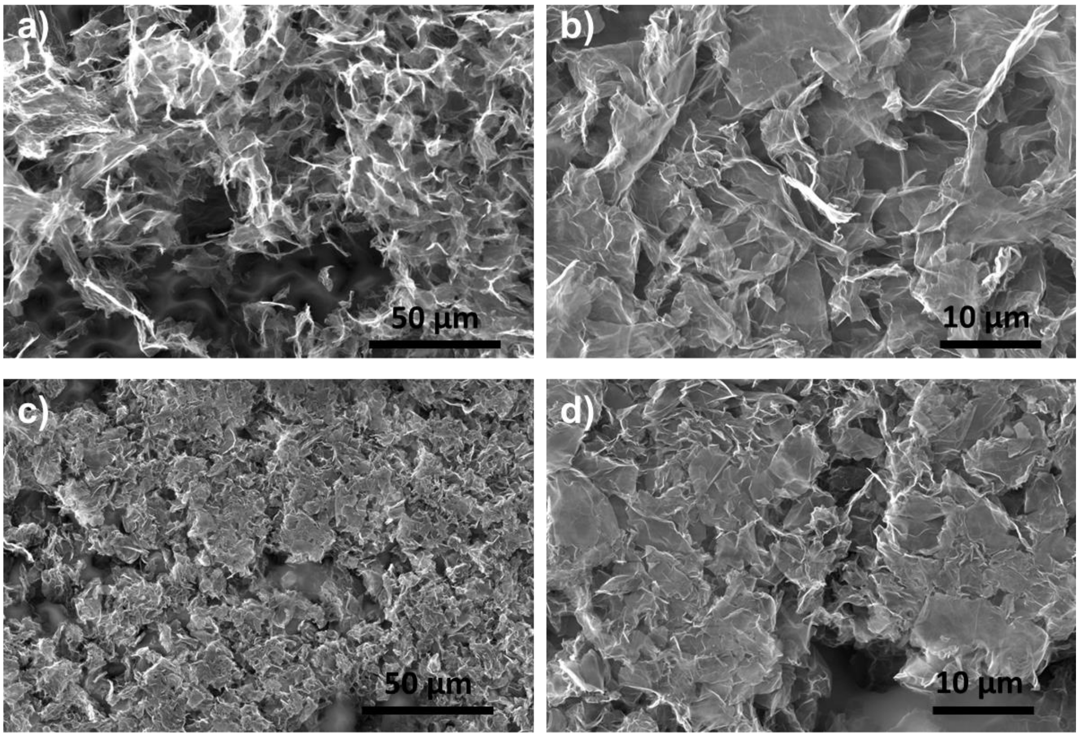

Figure S4. SEM images a-b) CrGO powders, and c-d) rGOSH powders.

4. EDX characterization

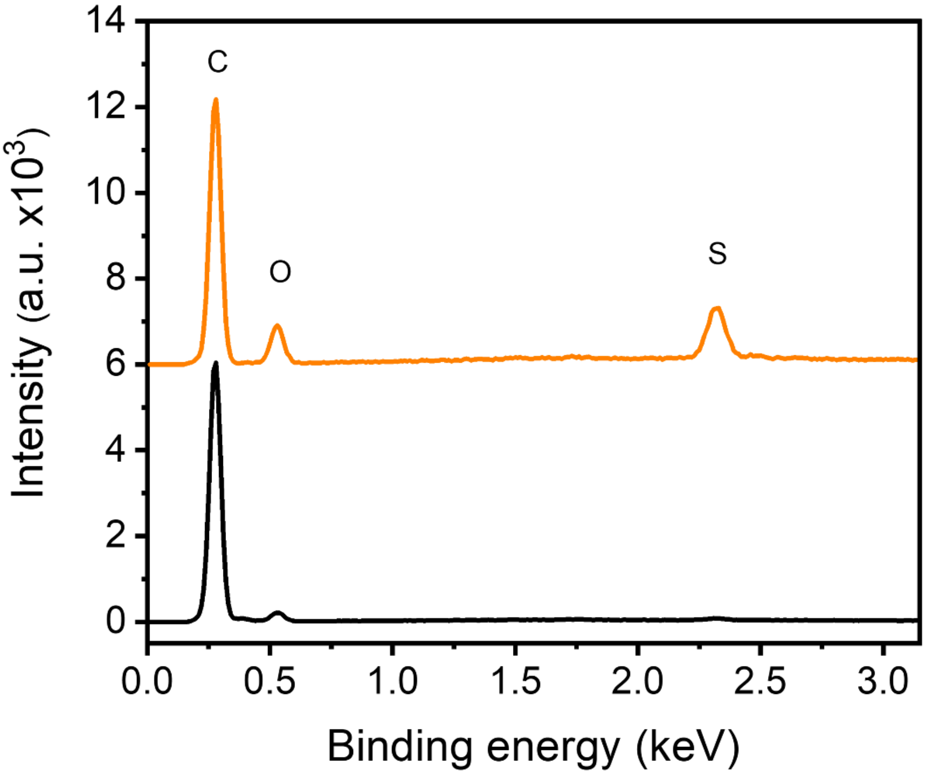

Figure S5. EDX spectra of CrGO (black line) and rGOSH (orange line)

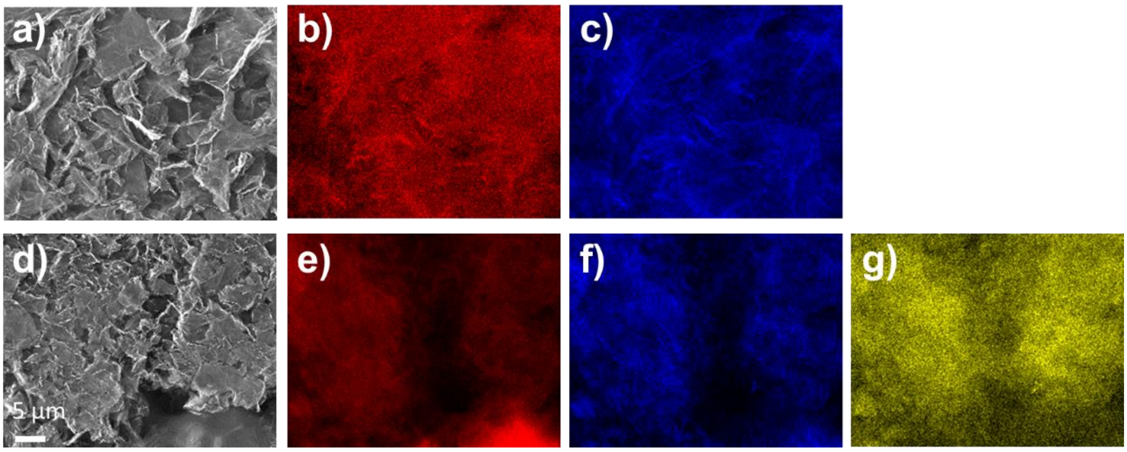

Figure S6. SEM images of a) CrGO and d) rGOSH. EDX mapping of element distribution in CrGO: b) carbon, and c) oxygen; EDX mapping of element distribution in rGOSH: e) carbon, f) oxygen, and g) sulfur.

5. Raman characterization

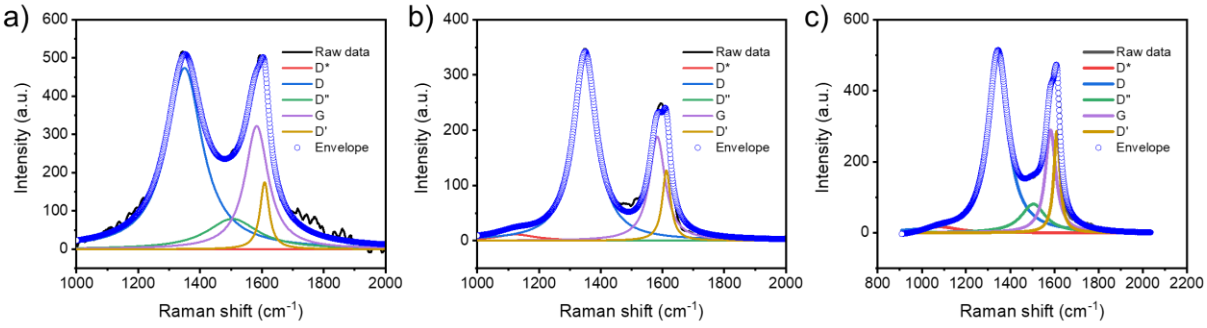

Figure S7. Fitted Raman spectra of a) GO, b) CrGO, and c) rGOSH

Table S1. $I_{D/G}$, $I_{D'/G}$, $I_{D''/G}$, and $I_{D*/G}$ ratio for CrGO and rGOSH

Ratio	GO	CrGO	rGOSH
$I_{D/G}$	1.47	1.80	1.75
$I_{D'/G}$	0.54	0.68	0.99
$I_{D''/G}$	0.25	0.06	0.28
$I_{D*/G}$	-	0.06	0.06

6. PXRD characterization

PXRD diffractograms have been recorded using a D8 Advanced with twin-twin optics (Bruker). The diffraction patterns of the powder sample have been acquired in 5-40 (2θ) range in Bragg-Brentano configuration, using $Cu_{K\alpha}$ radiation and a LINXEYE 2 detector. Two 2.5 mm Soller Cu slits have been applied to the primary and secondary optics. In the latter, a Ni stopper has been added to filter $Cu_{k\beta}$ radiations. An automatized blade has been mounted to limit the contribution of air scattering at low angles. The data has been acquired with a step of 0.02° and an acquisition time of 0.2 s per step.

Calculation of the XRD parameters

From the XRD diffractograms the peak position has been calculated using the Bragg's law:

$$d_{(hkl)} = \frac{\lambda}{2\sin\theta} \tag{1}$$

Where $d_{(hkl)}$ is the calculated inter planar distance (Å), λ is the wavelength of the XRD source (Å), θ is the scattering angle (rad). In the present case λ=1.54 Å.

The crystallite dimension has been derived from the Scherrer formula:

$$L_C = \frac{K\lambda}{\beta\cos\theta} \tag{2}$$

Where Lc is the crystallite thickness (Å), K is the shape factor equal to 0.89,[1] β is the FWHM of the (002) peaks and θ is the corresponding scattering angles.

The number of layer has been obtained from the following formula:

$$n_C = \frac{L_C}{d_{(hkl)}} \tag{3}$$

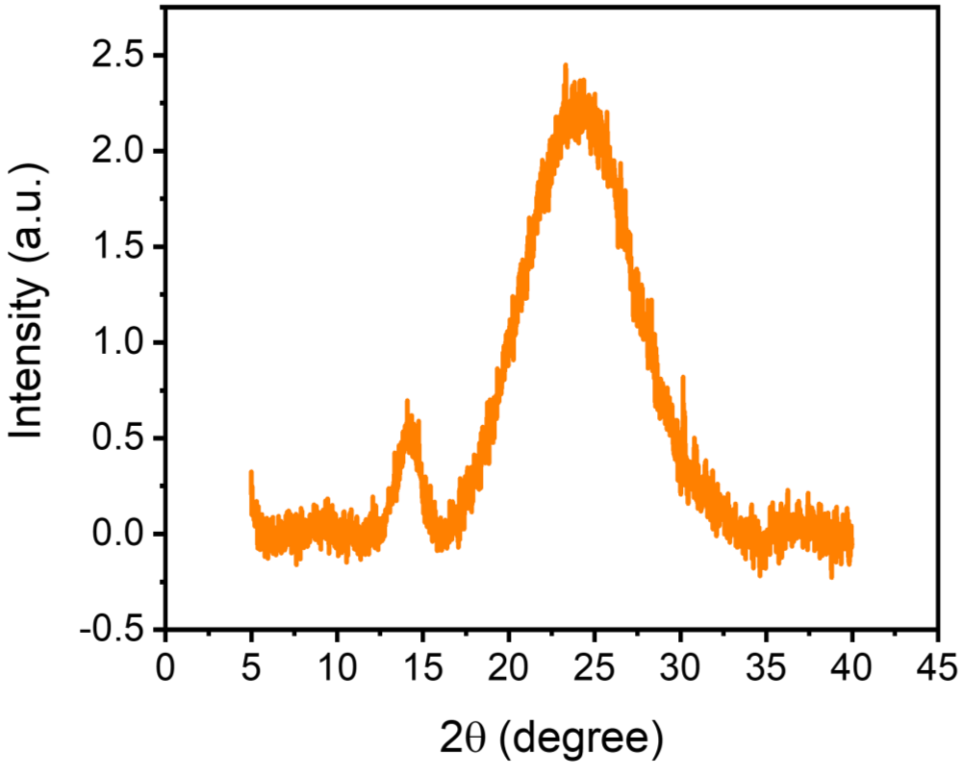

Figure S8. Diffraction pattern of rGOSH.

Table S2. The XRD peak position, d-spacing, crystallite thickness (L_c) and average graphene layer number (n_c) calculated for the (002) plane for GO, CrGO and rGOSH.[2]

**Sample**	**(0 0 2) position (°)**	***d*-spacing (Å)**	**L_c (Å)**	**Number of layers n_c**
GO	10.05	8.79±0.01	97.32±3.05	11.07
CrGO	23.34	3.81±0.01	14.19±0.71	3.73
rGOSH	24.27	3.66±0.01	10.83±0.43	2.96

7. BET characterization

Table S3. Physical parameters of GO, CrGO and rGOSH.

**Parameter**	**GO**	**CrGO**	**rGOSH**
**Surface area (m^2/g)**	12.61[3]	124.92[3]	78.96
**Average pore size (nm)**	6.95[3]	6.40[3]	10.20
**Electrical conductivity (S/m)**	-	3848	60
**Crystalline**	No	Yes	Yes
**Active sites**	C=O + C-OH	C=O + C-OH	C=O + C-OH + SH

8. Electrochemical characterization

Cyclic voltammetry

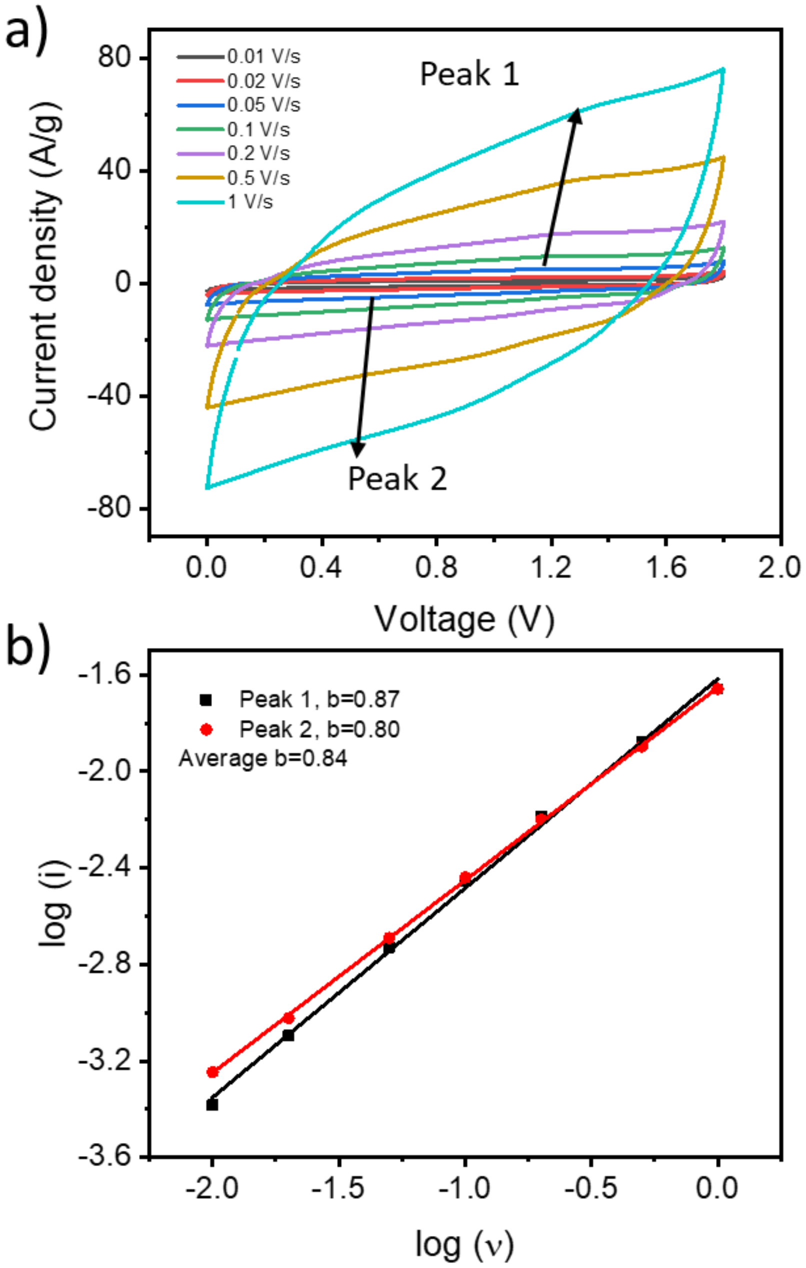

Figure S9. a) CV curves of CrGO at various scan rates, b) fitting plots between log(i) and log (υ) at various peak currents.

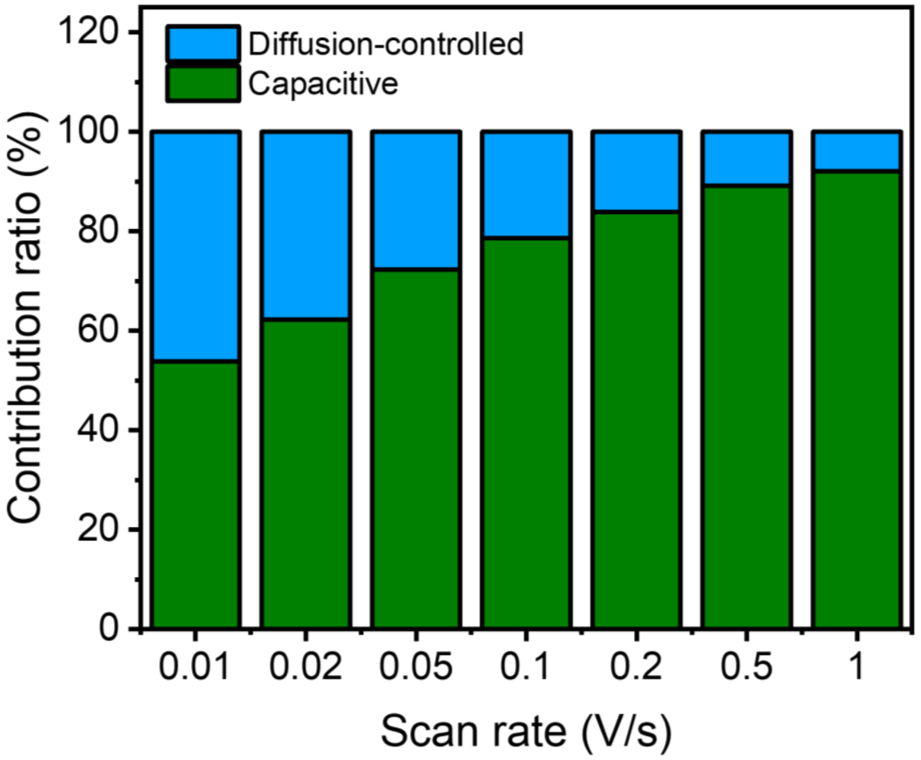

Figure S10. Capacitive (contribution) and diffusion-controlled contribution of CrGO at various scan rates

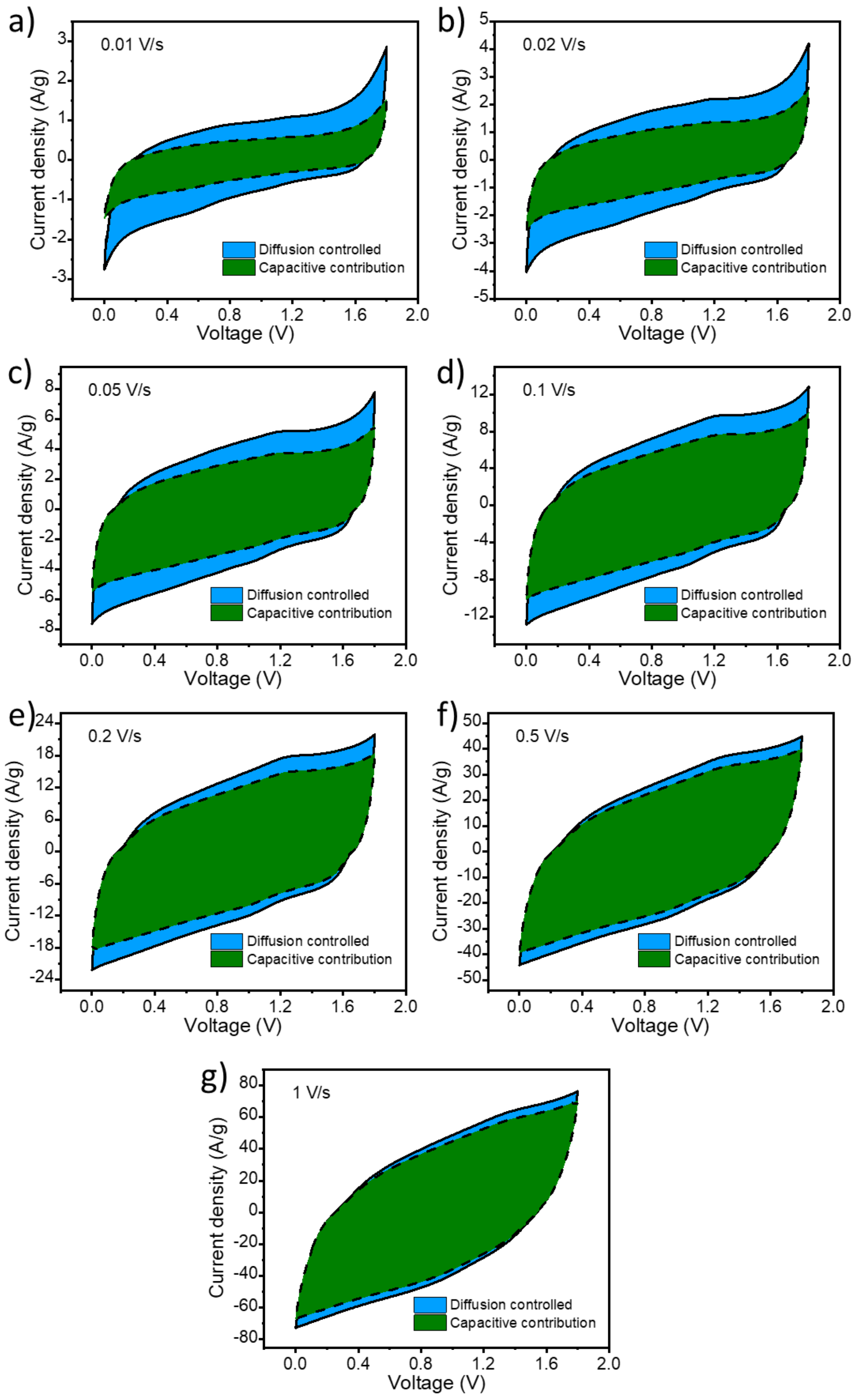

Figure S11. Capacitive (contribution) and diffusion-controlled contribution fraction for the CV curves of CrGO recorded at different scan rates.

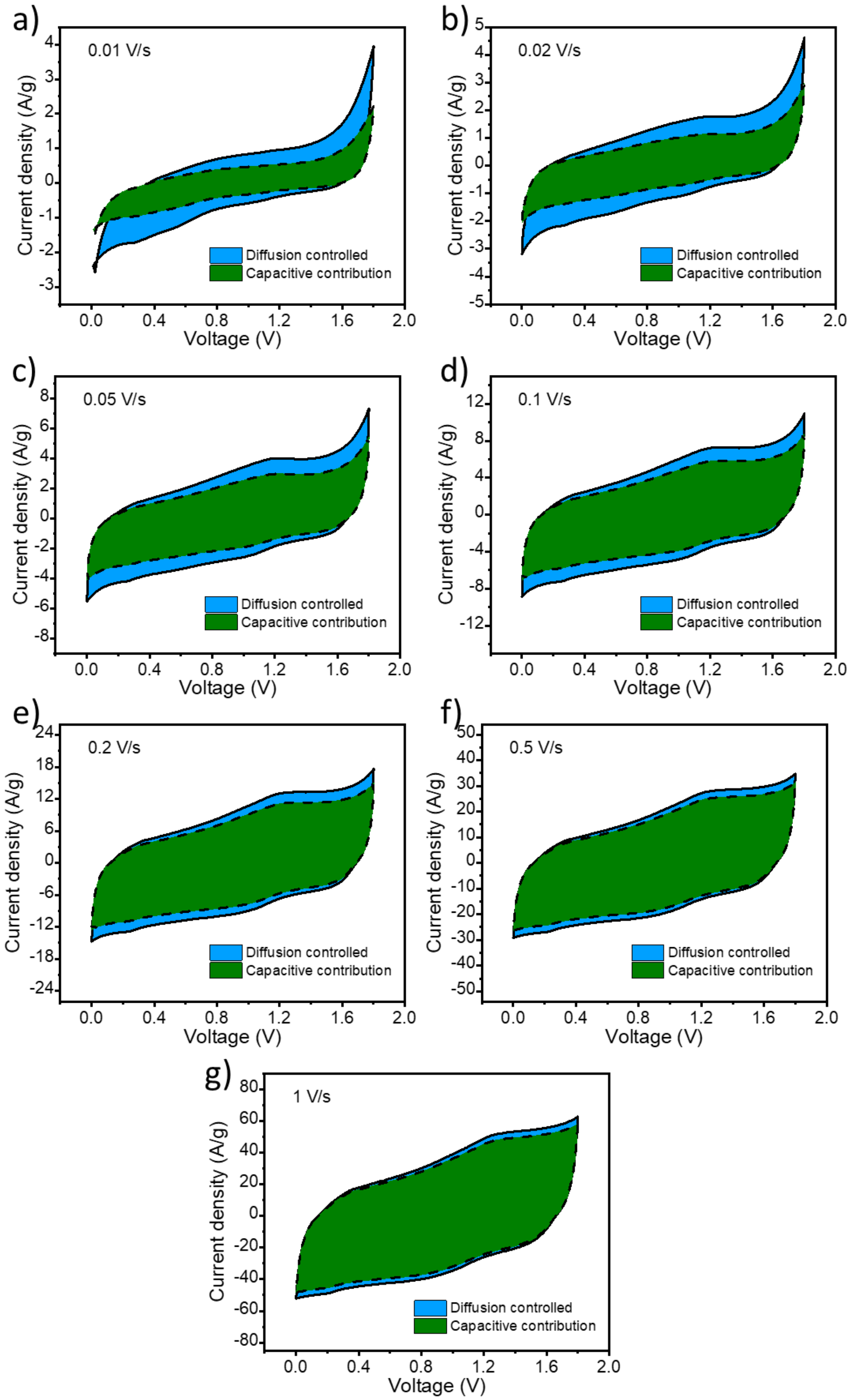

Figure S12. Capacitive (contribution) and diffusion-controlled contribution fraction for the CV curves of rGOSH recorded at different scan rates.

Electrochemical Impedance Spectroscopy (EIS)

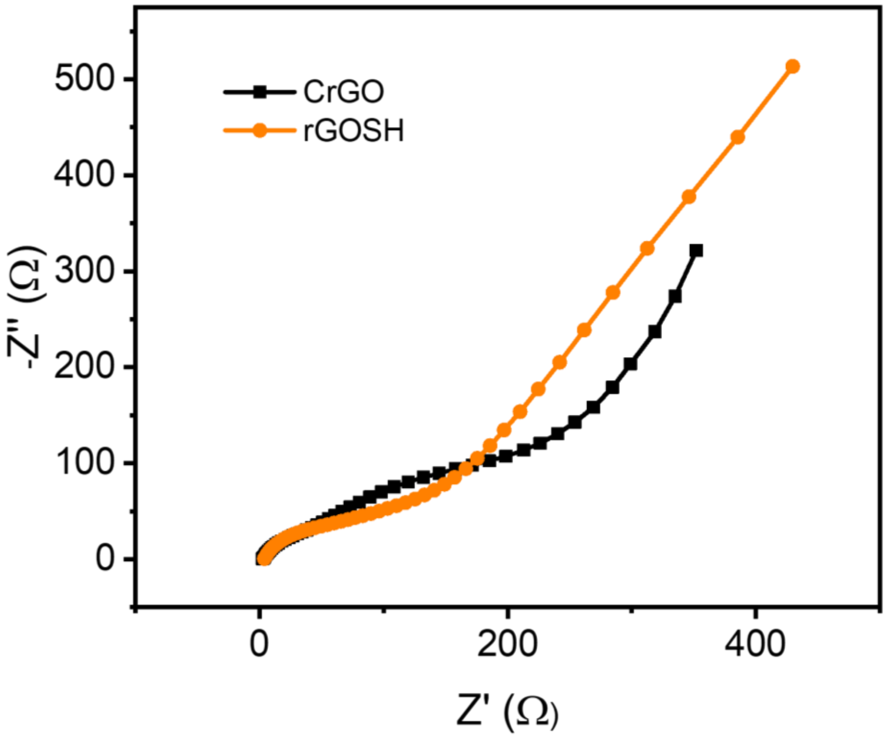

Figure S13. Nyquist plots of CrGO (black curve) and rGOSH (orange curve).

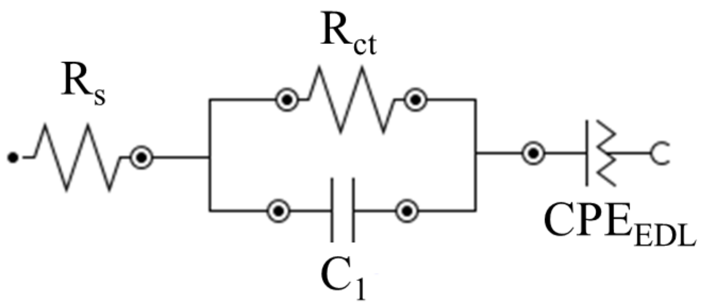

Figure S14. The equivalent electric circuit models used for fitting the Nyquist plots. R_s: the intrinsic ohmic resistance; R_{ct}: charge transfer resistance; C_1: capacitance element; CPE_{EDL}: constant phase element representing the electrical double layer capacitance (EDLC).

Table S4. Fitting parameters obtained from the Nyquist plots.

**Sample**	Rs (Ω)	Rct (Ω)
CrGO	2.17	23.59
rGOSH	5.90	46.41

Galvanostatic charge discharge

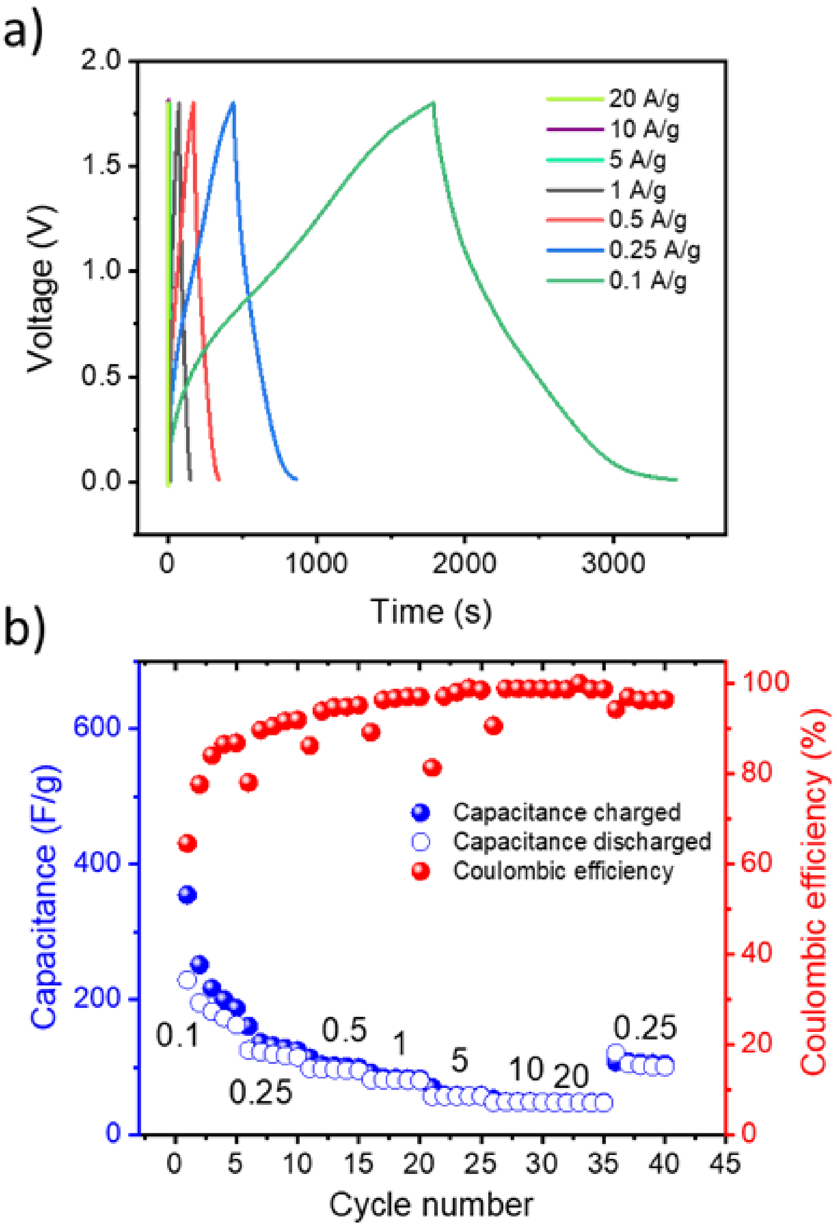

Figure S15. a) GCD profiles of CrGO at various current densities, b) specific capacitance (blue points), discharged (white points) and coulombic efficiency (red points) of CrGO at various current densities. Each current density is expressed in A/g.

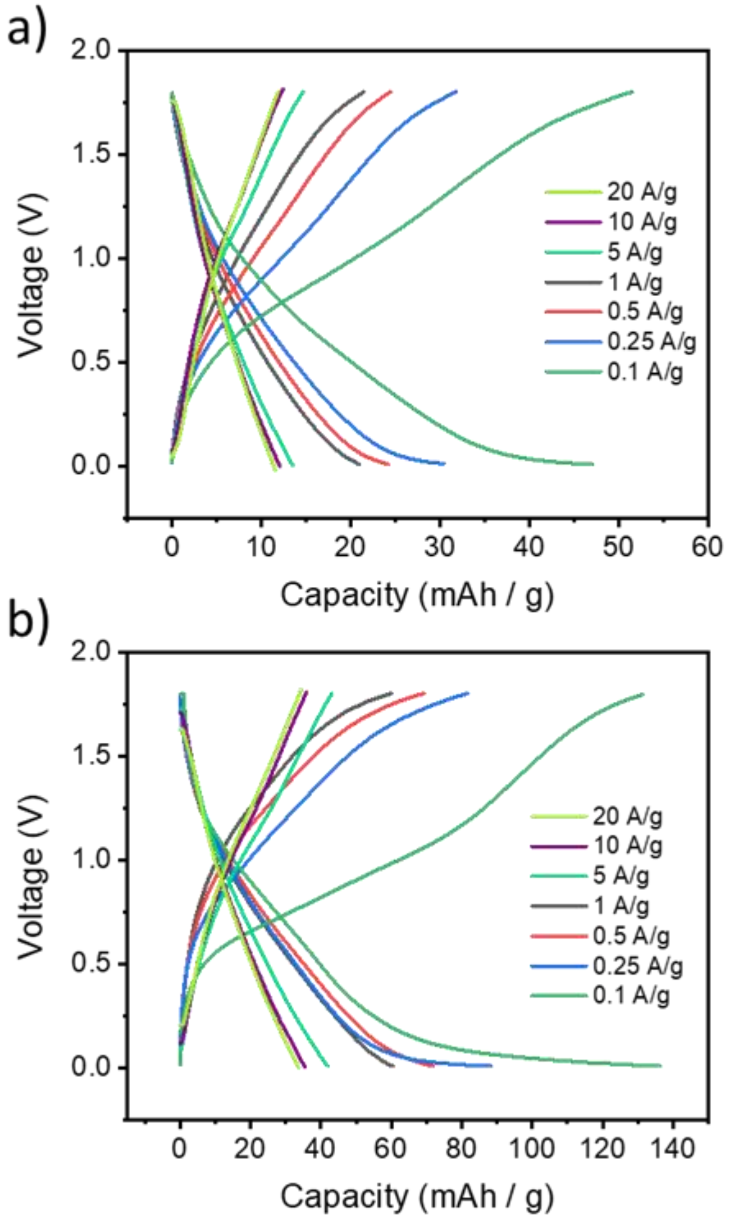

Figure S16. Specific capacity vs voltage for a) CrGO, and b) rGOSH at different current densities.

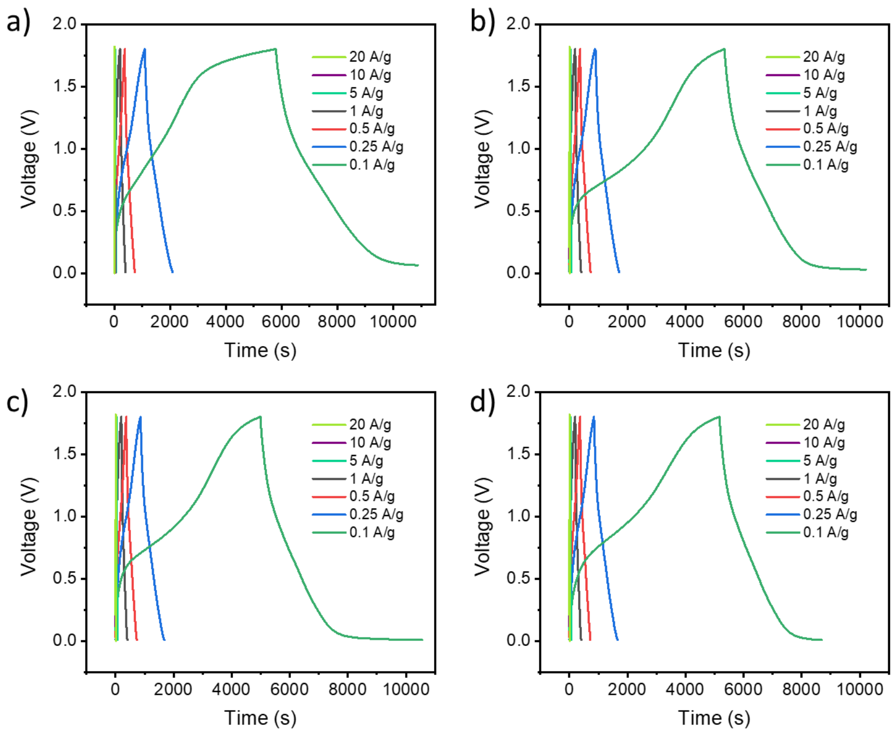

Figure S17. GCD profiles corresponding to four different samples of rGOSH at various current densities.

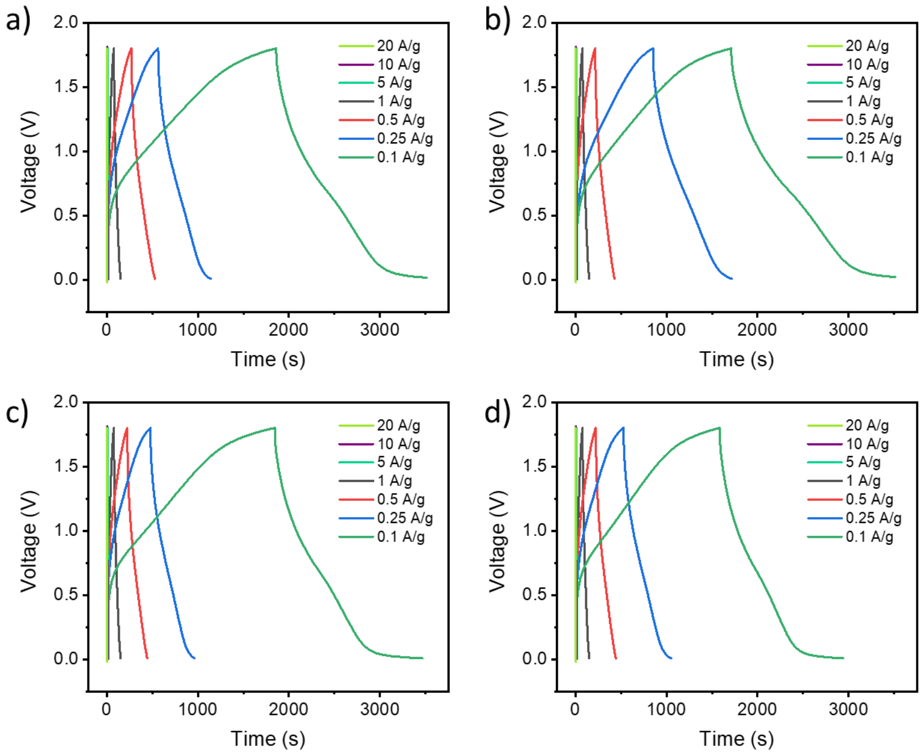

Figure S18. GCD profiles corresponding to four different samples of CrGO at various current densities.

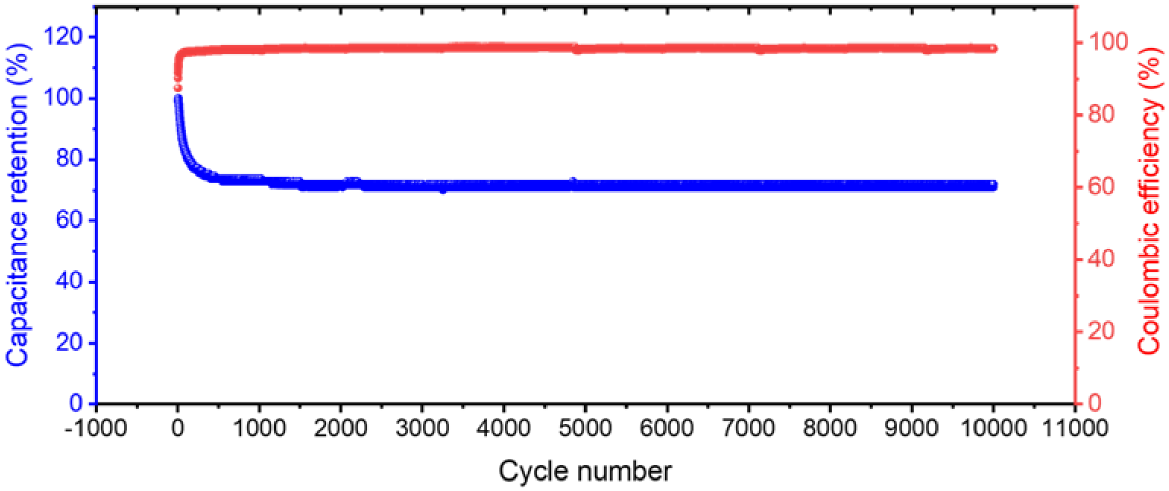

Figure S19. Long-term cycling performance of CrGO at 1 A/g.

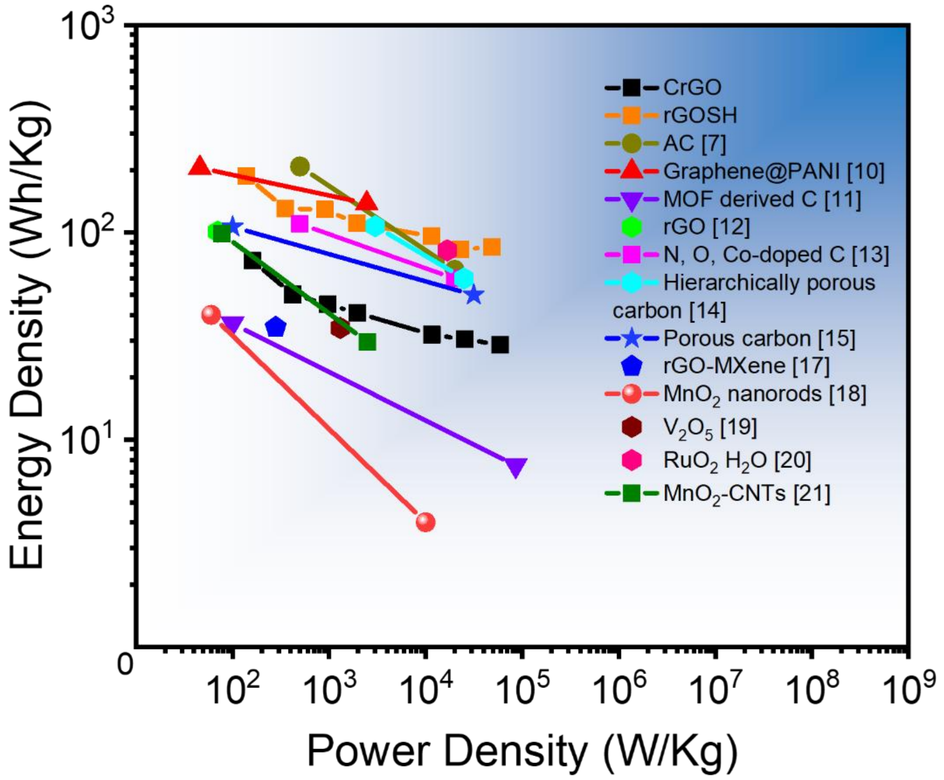

Figure S20. Ragone plot for CrGO (black points) and rGOSH (orange points) as well as other cathode materials employed in Zn-HSCs.

9. Charge – Discharge Mechanism investigation

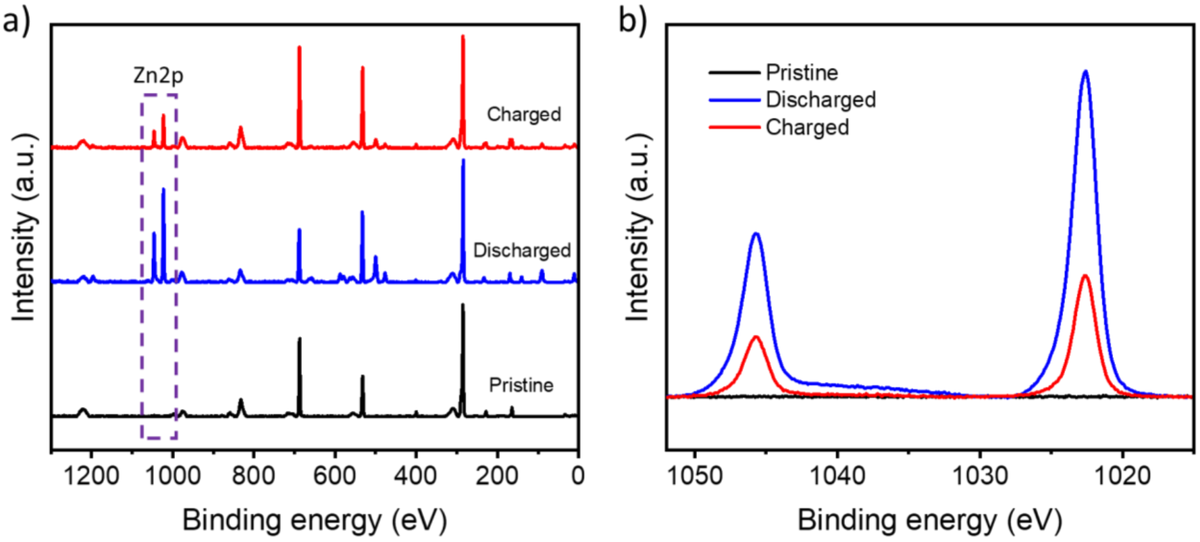

Figure S21. a) Survey (normalized by C1s) and b) Zn2p high resolution XPS spectra of rGOSH in pristine, discharged and charged form.

10. Computational methods

Optimized geometries and energy values were calculated using the semi-empirical method using Gaussian09 program.

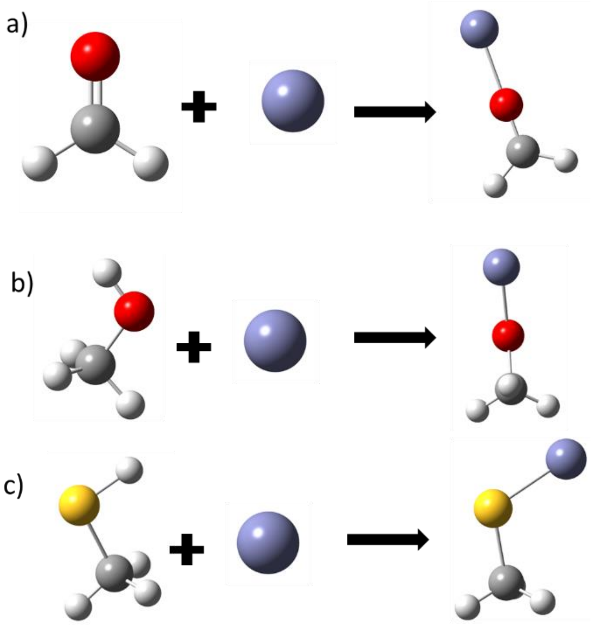

Figure S22. Chemical structures employed for the binding energy calculations.

Table S5. Parameters obtained from Gaussian for the binding energy calculations of carbonyl groups with Zn ions.

**H_3C-C=O structure**		
**System**	**Electronic E (au)**	**Electronic E (kcal/mol)**
H_3C-C=O	-0.03292263	-20.62
Zn	0.04967256	31.17
H_3C-C-O-Zn	0.00157012	0.99
**Binding energy**	-0.01517981	-9.53

Table S6. Parameters obtained from Gaussian for the binding energy calculations of hydroxyl groups with Zn ions.

**H_3C-C-OH structure**		
**System**	**Electronic E (au)**	**Electronic E (kcal/mol)**
H_3C-C-OH	-0.07693174	-48.27
Zn	0.04967256	31.17
H_3C-C-O-Zn	-0.05750366	-36.08
**Binding energy**	-0.03024522	-18.98

Table S7. Parameters obtained from Gaussian for the binding energy calculations of thiol groups with Zn ions.

**H_3C-S-H structure**		
**System**	**Electronic E (au)**	**Electronic E (kcal/mol)**
H_3C-SH	-0.00536540	-3.37
Zn	0.04967256	31.17
H_3C-S-Zn	-0.25917250	-162.63
**Binding energy**	-0.30347966	-190.44

Table S8. State of the art of the electrochemical performance of various cathode materials in Zn-HSCs.

Cathode	Anode	Electrolyte	Voltage window (V)	Capacitance (F/g)	Capacity (mAh/g)	Cyclability	Energy density (Wh/kg)	Power density (kW/kg)	Surface area (m^2/g)	Reference
rGOSH	Zn foil	4 M Zn $(CF_3SO_3)_2$	0-1.8	541 (0.1 A/g)	137 (0.1 A/g)	92% (10000 cycles)	187.6	48.61	78.96	This work
CrGO	Zn foil	4 M Zn $(CF_3SO_3)_2$	0-1.8	260 (0.1 A/g)	67.6 (0.1 A/g)	65% (10000 cycles)	73.2	59.15	124.92	This work
N,S co-doped G/PA	Zn foil	1 M $ZnSO_4$	0-1.8	268.4 (0.1 A/g)	-	93% (10000 cycles)	95.4	80	-	[4]
N,P co-doped rGO	Zn foil	1 M $ZnSO_4$	0-1.8	210.2 (1 A/g)	-	82% (15000 cycles)	94.6	0.45	-	[5]
HHT-rGO	Zn foil	1 M $ZnSO_4$	0-1.8	277 (0.1 A/g)	-	98% (20000 cycles)	-	-	-	[6]
AC	2D-Zn/Ni	1 M $ZnSO_4$	0.2-1.8	468 (0.5 A/g)	-	99% (10000 cycles)	208	20	2201	[7]
AC	Zn foil	2 M $ZnSO_4$	0.2-1.8	249 (0.05 A/g)	-	99% (10000 cycles)	115.4 (μWh/cm^{-2})	-	-	[8]
Oxydized carbon nanotubes	Zn foil	1 M $ZnSO_4$	0-1.8	53 (0.01 V/s)	-	100% (5000 cycles)	-	-	211	[9]

Graphene@PANI	Zn foil	2 M $ZnSO_4$	0.4-1.6	-	154 (0.1 A/g)	80.5% (6000 cycles)	138	2.45	-	[10]
MOF derived C	Zn foil	1 M $ZnSO_4$	0.1-0.7	134 (0.2 A/g)	-	99% (10000 cycles)	36.4	0.085	85.5	[11]
rGO	Zn foil	1 M $ZnSO_4$	0.2-1.6	370.8 (0.1 A/g)	-	94.5% (10000 cycles)	100.9	0.07	-	[12]
N, O, Co-doped C	Zn foil	2 M $ZnSO_4$	0.2-1.8	-	138.5 (0.5 A/g)	100% (10000 cycles)	110	20	197.45	[13]
Hierarchically porous carbon	Zn foil	1 M $ZnSO_4$	0-1.8	-	177.8 (4.2 A/g)	99.7% (20000 cycles)	107.3	24.9	2762	[14]
Porous carbon	Zn foil	3 M Zn $(CF_3SO_3)_2$	0-1.9	210 (0.1 A/g)	-	93% (80000 cycles)	106	31.4	2957	[15]
CNT delaminated V_2C MXene	Zn foil	1 M $ZnSO_4$	0.1-1.1	256.6 (1 A/g)	-	100% (4000 cycles)	-	-	61.18	[16]
rGO-MXene	Zn foil	2 M $ZnSO_4$	0.2-1.6	129 (0.4 A/g)	-	95% (75000 cycles)	35	4	-	[17]
MnO_2 nanorods	Zn foil	2 M $ZnSO_4$	0-2	-	54.1 (0.1 A/g)	65.3 % (3000 cycles)	34.8	0.1	1906	[18]
V_2O_5	AC	2 M $ZnSO_4$	0-2	-	58 (0.1 A/g)	97.3 % (6000 cycles)	28	1	1961	[19]

RuO_2 H_2O	Zn foil	1 M Zn $(CF_3SO_3)_2$	0.4-1.6	-	122 (1 A/g)	87.5 % (10000 cycles)	82	16.74	57	[20]
MnO_2-CNTs	Mxenes film	2 M $ZnSO_4$ + 0.1 M $MnSO_4$	0.8-1.9	115.1 (0.001 V/s)	-	83.6% (15000 cycles)	98.7	0.077	-	[21]
$VOPO_4$	Zn foil	1 M Zn $(CF_3SO_3)_2$	0-2	-	60 (0.05 A/g)	87% (600 cycles)	96	-	-	[22]

AC: activated carbon
PANI: polyaniline
CNT: carbon nanotubes
MOF: metal organic framework
C: carbon

11. References

[1] D. J. Lim, N. A. Marks, M. R. Rowles, *Carbon* **2020**, *162*, 475-480.

[2] C. Valentini, V. Montes-García, P. A. Livio, T. Chudziak, J. Raya, A. Ciesielski, P. Samorì, *Nanoscale* **2023**, *15*, 5743-5755.

[3] T. Chudziak, V. Montes-García, W. Czepa, D. Pakulski, A. Musiał, C. Valentini, M. Bielejewski, M. Carlin, A. Tubaro, M. Pelin, P. Samorì, A. Ciesielski, *Nanoscale* **2023**, *15*, 17765-17775.

[4] T. Song, H. Hao, Y. Zhao, X. Wang, C. Li, W. Li, *J. Alloys Compd.* **2022**, *924*, 166493.

[5] Y. Zhao, H. Hao, T. Song, X. Wang, C. Li, W. Li, *J. Power Sources* **2022**, *521*, 230941.

[6] Y. Shao, Z. Sun, Z. Tian, S. Li, G. Wu, M. Wang, X. Tong, F. Shen, Z. Xia, V. Tung, J. Sun, Y. Shao, *Adv. Funct. Mater.* **2021**, *31*, 2007843.

[7] G.-H. An, J. Hong, S. Pak, Y. Cho, S. Lee, B. Hou, S. Cha, *Adv. Energy Mater.* **2020**, *10*, 1902981.

[8] P. Zhang, Y. Li, G. Wang, F. Wang, S. Yang, F. Zhu, X. Zhuang, O. G. Schmidt, X. Feng, *Adv. Mater.* **2019**, *31*, 1806005.

[9] Y. Tian, R. Amal, D.-W. Wang, *Front. Energy Res.* **2016**, *4*.

[10] J. Han, K. Wang, W. Liu, C. Li, X. Sun, X. Zhang, Y. An, S. Yi, Y. Ma, *Nanoscale* **2018**, *10*, 13083-13091.

[11] T. Xiong, Y. Shen, W. S. V. Lee, J. Xue, *Nano Mater. Sci.* **2020**, *2*, 159-163.

[12] G. Sun, Y. Xiao, B. Lu, X. Jin, H. Yang, C. Dai, X. Zhang, Y. Zhao, L. Qu, *ACS Appl. Mater. Interfaces* **2020**, *12*, 7239-7248.

[13] X. Deng, J. Li, Z. Shan, J. Sha, L. Ma, N. Zhao, *J. Mater. Chem. A* **2020**, *8*, 11617-11625.

[14] H. Zhang, Q. Liu, Y. Fang, C. Teng, X. Liu, P. Fang, Y. Tong, X. Lu, *Adv. Mater.* **2019**, *31*, 1904948.

[15] S. Wu, Y. Chen, T. Jiao, J. Zhou, J. Cheng, B. Liu, S. Yang, K. Zhang, W. Zhang, *Adv. Energy Mater.* **2019**, *9*, 1902915.

[16] C. Wang, S. Wei, S. Chen, D. Cao, L. Song, *Small Methods* **2019**, *3*, 1900495.

[17] Q. Wang, S. Wang, X. Guo, L. Ruan, N. Wei, Y. Ma, J. Li, M. Wang, W. Li, W. Zeng, *Adv. Electron. Mater.* **2019**, *5*, 1900537.

[18] X. Ma, J. Cheng, L. Dong, W. Liu, J. Mou, L. Zhao, J. Wang, D. Ren, J. Wu, C. Xu, F. Kang, *Energy Storage Mater.* **2019**, *20*, 335-342.

[19] X. Ma, J. Wang, X. Wang, L. Zhao, C. Xu, *J Mater Sci Mater Electron* **2019**, *30*, 5478-5486.

[20] L. Dong, W. Yang, W. Yang, C. Wang, Y. Li, C. Xu, S. Wan, F. He, F. Kang, G. Wang, *Nanomicro Lett.* **2019**, *11*, 94.

[21] S. Wang, Q. Wang, W. Zeng, M. Wang, L. Ruan, Y. Ma, *Nanomicro Lett.* **2019**, *11*, 70.

[22] V. Vijayakumar, M. Ghosh, M. Kurian, A. Torris, S. Dilwale, M. V. Badiger, M. Winter, J. R. Nair, S. Kurungot, *Small* **2020**, *16*, 2002528.
